# Microglial DBP Signaling Mediates Behavioral Abnormality Induced by Chronic Periodontitis in Mice

**DOI:** 10.1002/advs.202406269

**Published:** 2024-10-21

**Authors:** Ting Cao, Dan Tian, Si‐Ying Wang, Yue Pan, Zhi‐Xuan Xia, Wei‐Kai Chen, Shao‐Wei Yang, Qing‐Quan Zeng, Yue‐Ling Zhao, Ling Zheng, Ning Li, Zhong‐Meng Lai, Yi‐Xiao Luo, Zu‐Cheng Shen

**Affiliations:** ^1^ Department of Children's Stomatology Stomatological Hospital of Xiamen Medical College, Xiamen Key Laboratory of Stomatological Disease Diagnosis and Treatment Xiamen 361003 China; ^2^ Department of Pharmacology School of Pharmacy Fujian Medical University Fuzhou 350122 China; ^3^ Department of Pharmacology, School of Basic Medicine and Life Science Hainan Medical University Haikou 571199 China; ^4^ Fujian Key Laboratory of Drug Target Discovery and Structural and Functional Research, School of Pharmacy Fujian Medical University Fuzhou 350122 China; ^5^ Department of Anesthesiology Union Hospital, Fujian Medical University Fuzhou 350001 China; ^6^ Hunan Province People's Hospital The First‐affiliated Hospital of Hunan Normal University Changsha 410002 China

**Keywords:** albumin D‐site‐binding protein, chronic periodontitis, depression‐like behaviors, hippocampus, microglia

## Abstract

Several lines of evidence implicate that chronic periodontitis (CP) increases the risk of mental illnesses, such as anxiety and depression, yet, the associated molecular mechanism for this remains poorly defined. Here, it is reported that mice subjected to CP exhibited depression‐like behaviors and hippocampal memory deficits, accompanied by synapse loss and neurogenesis impairment in the hippocampus. RNA microarray analysis disclosed that albumin D‐site‐binding protein (DBP) is identified as the most prominently upregulated target gene following CP, and in vivo and in vitro immunofluorescence methods showed that DBP is preferentially expressed in microglia but not neurons or astrocytes in the hippocampus. Interestingly, it is found that the expression of DBP is significantly increased in microglia after CP, and knockdown of microglial DBP ameliorated the behavioral abnormality, as well as reversed the synapse loss and hippocampal neurogenesis damage induced by CP. Furthermore, DBP knockdown improved the CP‐induced hippocampal inflammation and microglial polarization. Collectively, these results indicate a critical role of DBP in orchestrating chronic periodontitis‐related behavioral abnormality, hippocampal synapse loss and neurogenesis deficits, in which the microglial activation may be indispensably involved.

## Graphical abstract

This study provides direct evidence for chronic periodontitis (CP) increases the risk of mental illnesses and learning memory deficits. Integrating RNA microarray analysis and related behavioral tests, the study discloses that albumin D‐site‐binding protein (DBP) is significantly increased in microglia after CP, and knockdown of microglial DBP ameliorated the behavioral abnormality, these findings pave a promising strategy for CP‐related emotion and learning memory disorders treatment.

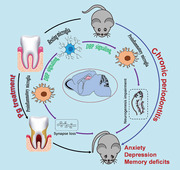

## 1 Introduction

Chronic periodontitis (CP), an inflammatory disease, which is characterized by formation of periodontal pockets, inflammation of pocket walls, absorption of alveolar bone and gradual loosening of teeth, and it is the main cause of tooth loss in adults.^[^ ^1^ ^]^ Recently, an epidemiological investigation shows that the probability of people suffering from varying degrees of periodontitis is over 80%, and this proportion is even higher among males and smokers.^[^ ^2^ ^]^ Importantly, there has been an increasing amount of evidence showing that CP is a possible risk factor for mental disorders, such as major depression disorders, anxiety and memory deficits.^[^ ^3^ ^]^ However, thus far, the molecular mechanisms by which CP mediates behavioral abnormality remain elusive.

Recently, an epidemiological survey in patients with mild periodontitis showed that the left hippocampus atrophies as the number of teeth present (NTP) decreases, while in patients with severe periodontitis, atrophy of the left hippocampus exacerbates as the NTP increases.^[^ ^4^ ^]^ In addition, the results of animal experiments also showed that periodontitis could induce cognitive function impairment and dendritic spine immaturity, and the possible cause is the activation of hippocampal microglia and neuroinflammation.^[^ ^5^ ^]^ Furthermore, CP exacerbated cognitive impairment in 5xFAD mice, with the deposition of amyloid plaque and decreased number of neuroprotective microglia in the hippocampus.^[^ ^6^ ^]^ These results suggest that CP may induce behavioral abnormality via the activation of hippocampal microglia, and then the associated neuroinflammation mediated synapse loss in the hippocampus. Besides synapse loss, neurogenesis impairment may also be a potential mechanism for CP‐ induced behavioral abnormality.^[^ ^7^ ^]^ However, the mechanisms underlying the effect of CP on synapse loss and neurogenesis impairment in the hippocampus are not well elucidated.

Albumin D‐box binding protein, also known as albumin D‐site‐binding protein (DBP), belongs to the proline and acidic amino acid‐rich basic leucine ZIPper family of transcription factors.^[^ ^8^ ^]^ DBP was originally described as a factor binding to the albumin D‐site, and known to govern circadian transcription of a number of hepatic detoxification and metabolic enzymes.^[^ ^9^ ^]^ Interestingly, DBP expression is almost undetectable in the morning and peaks at approximately 8:00 PM in rat liver.^[^ ^10^ ^]^ So far, the functional research of DBP has focused on the regulation of metabolic disorders, oxidative stress, hormone receptor abnormalities, and other disease processes in peripheral tissues. For example, DBP was reported to participate in the progression of mesangial proliferative nephritis through regulating the expression of inflammatory cytokines expression.^[^ ^11^ ^]^ Another study also showed that DBP plays an important role in regulating human TH9 differentiation in vitro.^[^ ^12^ ^]^ In addition, early research results indicate that DBP is also expressed in the central nervous system, such as the hippocampus and suprachiasmatic nucleus, and DBP‐deficient mice do not exhibit major abnormalities,^[^ ^13^ ^]^ while triple mutant mice lacking DBP develop lethal spontaneous seizures associated with dysregulation of neurotransmitter homeostasis in the brain.^[^ ^14^ ^]^ Moreover, DBP is also known as a key age‐associated regulator of the neuronal transcription of tissue‐type plasminogen activator (tPA), and inhibition of DBP‐mediated tPA expression confers in vitro neuroprotection.^[^ ^15^ ^]^ Behaviorally, DBP overexpression inhibited spatial learning but not memory, and enhanced susceptibility to kainate‐induced seizures.^[^ ^16^ ^]^ To date, little is known about DBP underlying spatial learning, and it remains unclear whether there is a causal relationship between DBP and neuronal proliferation and neurogenesis in the brain, particularly the behavioral abnormality induced by CP.

In this study, to investigate the mechanism of abnormal behaviors induced by CP in mice, we established the CP model using *Porphyromonas gingivalis* (Pg), and detected the anxiety‐like behaviors by elevated plus maze (EPM) and open field test (OFT), depression‐like behaviors by sucrose preference test (SPT), tail suspension test (TST) and forced swimming test (FST), and learning memory by Morris water maze, Barnes maze and fear conditioning experiments. Then, we examined the hippocampal synapse loss and neurogenesis by immunofluorescence assay, and found there were obvious synapse loss and neurogenesis impairment in the hippocampus. Furthermore, high throughput sequencing was used to screen the targets which may mediate the behavioral abnormality induced by CP. We found that DBP was identified as the most prominently upregulated gene, and in vivo and in vitro results showed that DBP was prominently stained in microglia but not neurons or astrocytes. Lastly, DBP was selectively knockdown in microglia by recombinant adeno‐associated virus, and we found that DBP knockdown ameliorated the behavioral abnormality, and reversed the hippocampal synapse loss and damage to neurogenesis induced by CP. Therefore, our results reveal previously unknown roles of DBP in behavioral abnormality induced by CP through regulation of microglial phenotype.

## 2 Results

### 2.1 CP Induces Anxiety‐ and Depression‐Like Behaviors in Mice

**Figure 1:**
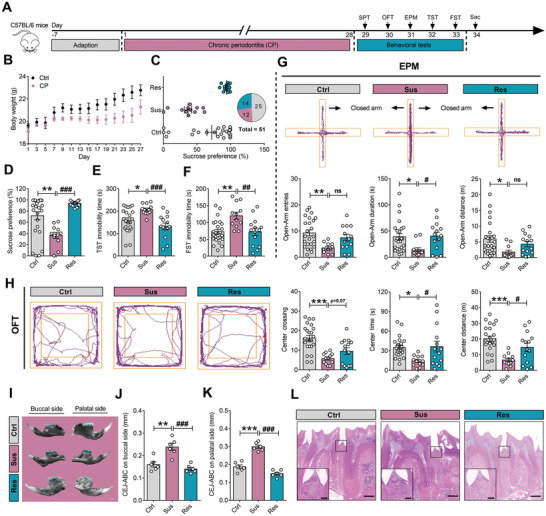
Chronic periodontitis induces anxiety‐ and depression‐like behaviors in mice. A) Experimental schedule of chronic periodontitis (CP) modeling and tests of emotional behaviors. B) Changes of the average body weight during the 28‐days CP modeling. C‐D) The sucrose preference of Ctrl mice and Sus or Res mice subjected to CP in SPT. E‐F) The immobility time of Ctrl, Sus, and Res mice in TST (E) and FST (F). G) The movement traces, open‐arm entries, open‐arm duration, and open‐arm distance of Ctrl, Sus, and Res mice in EPM. H) The movement traces, center crossing times, center duration, and center distance of Ctrl, Sus, and Res mice in OFT. I) The buccal and palatal structure of teeth and periodontal tissue from Ctrl, Sus, and Res mice. J‐K) The statistical results of CEJ‐ABC on buccal J) and palatal K) sides of Ctrl, Sus, and Res mice. L) The representative H&E staining of periodontal tissues from Ctrl, Sus, and Res mice. All data are presented as mean ± SEM. ns, no significant difference. Student's t‐test for (B), two‐way ANOVA for (D‐F, G, H, J, K). n = 25, 12, 14 per group for (C‐H); n = 6 per group for (J, K). *p < 0.05, **p < 0.01 and ***p < 0.001 versus Ctrl group; ^#^ *p* < 0.05, ^##^ *p* < 0.01 and ^###^ *p* < 0.001 versus Sus group.

CP has been demonstrated to be involved in behavioral abnormality in human.^[^ ^17^ ^]^ However, there is no direct evidence to show the effects and mechanisms of CP on anxiety‐ and depression‐like behaviors in mice. To explore this, mice were administrated with Pg suspension via oral gavage four times per day for one month, and at the end of CP modeling, anxiety‐ and depression‐like behaviors were assessed by EPM, OFT, SPT, TST, and FST tests (**Figure** **1**A). First, we observed that there was a significant lowness in weight of mice in the CP group when compared with the control (Ctrl) group (Figure 1B). Then, according to sucrose preference in SPT, CP mice were separated into susceptible and resilient subpopulations (Figure 1C). Mice with sucrose preference ratio significantly lower than Ctrl group were defined as susceptible, while mice with higher sucrose preference ratio were identified as resilient. For the Ctrl group in this study, the average sucrose preference ratio was 71.8%, mice with sucrose preference ratio lower than Ctrl group were defined as the susceptible group, while mice with higher sucrose preference ratio were identified as the resilient group. As the results shown, susceptible mice displayed a significant decrease in sucrose preference (Figure 1D), as well as increased immobility time in TST and FST (Figure 1E,F). Furthermore, we also detected the anxiety‐like behaviors in mice by EPM and OFT, and found that compared with the Ctrl group, susceptible mice displayed decreased open arm exploration in the EPM (Figure 1G), including decreased open arm duration, open arm entries and open arm distance, and the reduced open arm exploration was not due to a decrease in locomotor activity, as demonstrated by the similar moving speed (Figure S1A, Supporting Information). As for the anxiogenic effect, similarly, susceptible mice spent less time in the center area (Figure 1H), without differences in moving speed in the OFT (Figure S1B, Supporting Information). These results demonstrate that CP could induce anxiety‐ and depression‐like behaviors in mice.

Next, to further examine the causal relationship between CP and anxiety‐/depression‐like behaviors, we detected the distance from the cement enamel junction (CEJ) to the alveolar bone crest (ABC) at nine predetermined sites in the maxilla on the buccal and palatal surfaces in three groups of mice. We found that there were significant and stable increases in dental root exposure and diastema on both the palatal and buccal surfaces of the left upper molars in susceptible mice compared with the control and resilient mice (Figure 1I–K). Moreover, we detected the inflammatory cell infiltration by H&E staining, and the histological examination revealed that gingival tissues of the susceptible mice had more inflammatory cell infiltration compared with the control and resilient groups (Figure 1L). These findings indicate that CP with Pg infection caused bone loss and inflammation, as well as anxiety‐ and depression‐like behaviors in mice.

### 2.2 CP Induces Hippocampal Memory Deficits in Mice

**Figure 2:**
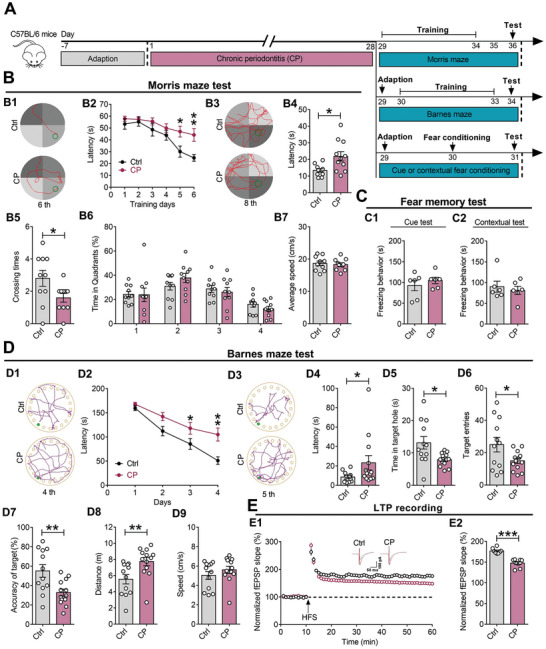
Chronic periodontitis induces hippocampal memory deficits in mice. A) Experimental schedule of CP modeling and behavioral tests related to learning and memory. B) Data analysis of Morris water maze test of Ctrl and CP mice, including the movement traces (B1, B3), the latency to the platform area (B2, B4) during training and testing, the platform crossing times (B5), the time spent in each quadrant (B6) and average speed (B7) on the probe trail at Day 8. C) The freezing time duration of Ctrl and CP mice in cue (C1) and contextual (C2) fear condition test at Day 3. D) Data analysis of Barnes maze test of Ctrl and CP mice, including the movement traces (D1, D3), the latency to target hole for the first time (D2, D4) during training and testing, the time spent nosing the target hole (D5), target hole entries (D6), accuracy of target hole (D7), distance traveled (D8) and average speed (D9) at Day 5. The accuracy of target hole is calculated as the ratio of time duration in target hole area/ the total time spent nosing the target and non‐target holes. E) Time‐course of LTP induced by HFS in hippocampal slices from the Ctrl and CP groups (E1), and the statistical results relative to baseline in Ctrl and CP groups (E2). All data are presented as mean ± SEM. Student's t‐test for all. n = 10 per group for (B); n = 6 per group for (C); n = 12, 13 per group for (D); and n = 10 per group for (E). *p < 0.05, **p < 0.01 and ***p < 0.001 versus Ctrl group.

Previous reports have revealed that CP may be relevant to processes associated with learning and memory, such as memory impairment and cognitive decline,^[^ ^18^ ^]^ and different brain regions were engaged in different memory. For example, hippocampal memory deficits are involved in Alzheimer's disease and aging‐related cognitive disorders,^[^ ^19^ ^]^ and post‐traumatic stress disorder was shown to be dependent on hippocampus and amygdala.^[^ ^20^ ^]^ However, which types of memory and which brain regions are related to CP is still unknown. To examine the effect of CP on learning and memory, we tested the associated behaviors by Morris water maze, Barnes maze, and fear condition following CP modeling (**Figure** **2**A). First, we evaluated spatial memory by Morris water maze task after CP, and observed that CP mice displayed longer latency from the fifth day during the learning stage (Figure 2B1,2). During the probe trial test, compared with the Ctrl mice, CP mice showed longer latency for the first crossing, fewer crossings to the platform region, and shorter swimming time in the target quadrant, with no changes in swimming speed (Figure 2B3‐7). Interestingly, in the fear conditioning test, CP mice showed no significant alterations in freezing time in the cue conditioning paradigms, which depends only on amygdala, as well as in the contextual associative fear memory, which relies on hippocampus and amygdala (Figure 2C1,2; Figures S2 and S3, Supporting Information). Furthermore, in the Barnes maze task, CP mice displayed fewer nose poke numbers within the target hole, longer latency to the target hole, and more errors made during the search stages for the target hole than the Ctrl mice (Figure 2D1‐9). Finally, our fEPSP recordings from hippocampal slices showed that CP mice displayed impairment in LTP because the normalized fEPSP slope was dramatically suppressed compared with the Ctrl group (Figure 2E1,2). Together, these results indicate that CP induced hippocampal synaptic disorder in vivo and memory deficits in mice.

### 2.3 CP Causes Synapse Loss and Neurogenesis Impairment in the Hippocampus

**Figure 3:**
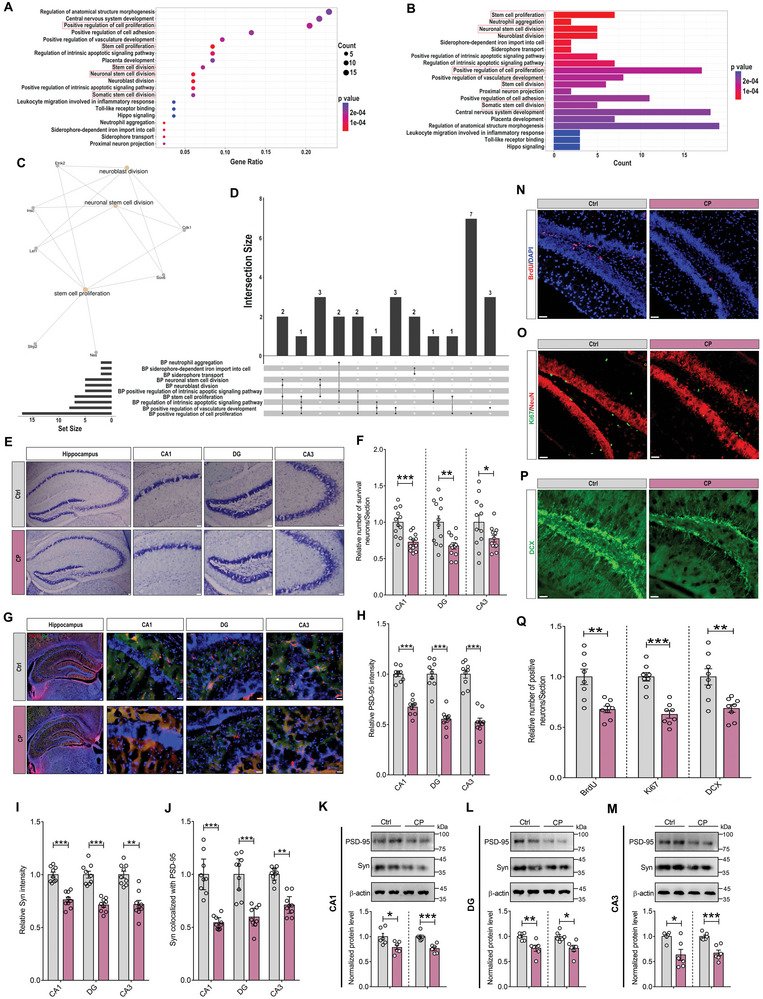
Chronic periodontitis induces synapse loss and neurogenesis impairment in the hippocampus. A) Gene ontology (GO) enrichment bubble map of differentially expressed genes (DEGs) between Ctrl and CP mice showing the top 20 enriched biological process terms. B) GO enrichment histogram of DEGs between Ctrl and CP mice showing the top 20 enriched biological process terms. C) GO functional enrichment network of DEGs between Ctrl and CP mice. D) GO enrichment upset map of DEGs between Ctrl and CP mice. E) Representative images of Nissl staining in the hippocampus of Ctrl and CP mice. Scale bar, 300 µm in left and 100 µm in enlarged images. F) Statistical results of Nissl's staining in the hippocampal sub‐regions of CP mice relative to Ctrl mice. G) Double‐staining of PSD‐95 (blue) and synaptophysin (green) in the hippocampus of Ctrl and CP mice. The right are magnified images showing the colocalization of PSD‐95 and synaptophysin. Scale bar, 300 µm in left and 100 µm in enlarged images. H‐J) Statistical results of PSD‐95 intensity (H), synaptophysin intensity (I) and colocalized ratio between PSD‐95 and synaptophysin (J) in the hippocampal sub‐regions of CP mice relative to Ctrl mice. K‐M) Representative Western blots and quantification of PSD‐95 and synaptophysin expression in the CA1 (K), DG (L), and CA3 (M) of CP mice relative to Ctrl mice. N) Representative BrdU‐ and DAPI‐staining of hippocampus from Ctrl and CP mice. Scale bar, 100 µm. O) Representative Ki67‐ and NeuN‐stained images of hippocampus from Ctrl and CP mice. Scale bar, 100 µm. P) Representative DCX‐stained images of hippocampus from Ctrl and CP mice. Scale bar, 100 µm. Q) Quantification of the relative number of BrdU‐, Ki67‐ or DCX‐ positive neurons in hippocampus from Ctrl and CP mice. All data are presented as mean ± SEM. Student's t‐test for all. n = 12 per group for (F); n = 9 per group for (H‐J); n = 6 per group for (K‐M); and n = 8 per group for (Q). *p < 0.05, **p < 0.01 and ***p < 0.001 versus Ctrl group.

Our above results suggest that hippocampus, not amygdala, may play a critical role in regulating the behavioral abnormalities induced by CP. However, the underlying mechanisms of these processes remain unclear. Therefore, firstly we collected the hippocampal tissues to perform RNA‐Seq analysis, and the results of gene ontology (GO) enrichment analysis suggested that CP may mediate behavioral abnormality by regulating the neuronal stem cell division and proliferation (**Figure** **3**A–D). To further validate the possible mechanisms, we examined the neuronal apoptosis by Nissl staining, and found that CP induced obvious neuronal apoptosis in the three sub‐regions of hippocampus (Figure 3E,F). Then, we tested synaptic markers to evaluate synapse loss in dentate gyrus (DG), CA1, and CA3 regions, such as synaptic vesicle proteins such as synaptophysin, and postsynaptic markers such as PSD‐95. The results of immunofluorescence assays showed that CP caused remarkable decreases in expression and the merge ratio of synaptophysin and PSD‐95. These results revealed that CP mediated remarkable synapse loss in the hippocampus (Figure 3G–J). In line with these findings, similar results were observed in the Western blotting detection (Figure 3K–M). Finally, neuronal stem cell division and proliferation were measured by BrdU, Ki67 and DCX staining, which were identified as the markers of dividing cells and immature neurons, respectively. It was found that the numbers of BrdU and Ki67 positive cells were both significantly declined in the DG of CP mice, and the number of DCX positive cells number was also decreased obviously (Figure 3N–Q). Taken together, these results suggest that CP may mediate behavioral abnormality by inducing synapse loss and neurogenesis impairment in the hippocampus.

### 2.4 Microglial DBP is Required for CP‐Induced Depression‐Like Behaviors and Memory Deficits

**Figure 4:**
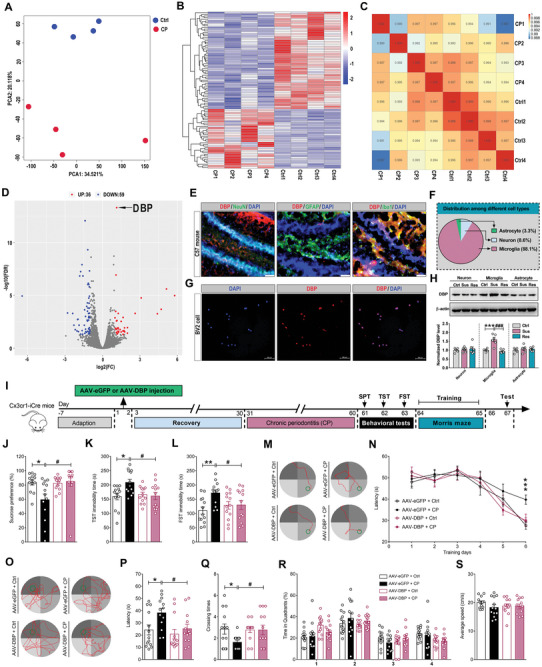
Microglial DBP signaling is required for depression‐like behaviors and memory deficits induced by chronic periodontitis. A) The principal component analysis (PCA) table showing the sample repeatability of Ctrl and CP mice. B) Clustering heat map of DEGs in the hippocampus of CP mice relative to Ctrl mice. C) Correlation matrix showing the correlation between samples of hippocampus from Ctrl and CP mice. D) Volcano map showing the number of DEGs in hippocampus between Ctrl and CP mice. E) Immunofluorescence of DBP colocalized with cell specific markers for neuron (NeuN), astrocyte (GFAP) and microglia (Iba1) in hippocampus of C57 mouse, respectively. Scale bar, 100 µm. F) Pie chart showing the distribution of DBP among astrocyte, neuron and microglia in the hippocampus. G) Immunofluorescence assay results showing the colocalization of DBP within BV2 cell cultured in vitro. Scale bar, 100 µm. H) Representative Western blots and quantification of DBP expression in the neuron, microglia, and astrocyte of hippocampus samples from Ctrl, Sus, and Res mice. I) Experimental schedule of viral microinjection in the hippocampus of Cx3cr1‐iCre mice, CP modeling, and tests of emotional behaviors and memory. J‐L) Effects of specific microglial DBP knockdown on depression‐like behaviors, such as sucrose preference in SPT (J), immobility time in TST (K) and FST (L). M) Representative movement traces of Morris water maze test on learning stage. N) Effects of specific microglial DBP knockdown on average latency to platform in Morris water maze test on learning stage from Day 1 to Day 6. O) Representative movement traces of Morris water maze test on the probe trail at Day 8. P‐S) Effects of specific microglial DBP knockdown on the latency of the first reach to the platform area (P), platform crossing times (Q), total time spent in different quadrants (R) and average speed (S) in Morris water maze test on Day 8. All data are presented as mean ± SEM. Two‐way ANOVA for all. n = 6 per group for (H), and n = 13 per group for (J‐L, N, P‐S). *p < 0.05, **p < 0.01 and ***p < 0.001 versus Ctrl group or AAV‐eGFP + Ctrl group. ^#^ *p* < 0.05, ^###^ *p* < 0.001 versus Sus group or AAV‐eGFP + CP group.

To investigate the regulating mechanism of CP on synapse loss and neurogenesis impairment in the hippocampus, transcriptome analysis of hippocampus from the Ctrl and CP mice was performed. Firstly, sample repeatability of the Ctrl and CP mice was shown in the principal component analysis (PCA) table (**Figure** **4**A), and the clustering heat map of DEGs and correlation matrix between the Ctrl and CP hippocampal tissues were also examined (Figure 4B,C). Then, we analyzed the DEGs using a log_10_ fold change ≥ 2.0 and a P ≤ 0.01, and found that only 36 genes were upregulated, and 59 genes were downregulated in the hippocampus of the CP mice. Among the upregulated genes, D‐site‐binding protein, also known as DBP, was identified as the most prominently upregulated gene in the CP mice (Figure 4D). Furthermore, we examined the distribution of DBP with cell specific markers for neuron (NeuN), astrocyte (GFAP), and microglia (Iba1) respectively in hippocampus of C57 mice. Interestingly, we found that DBP preferentially colocalized with Iba1 and the co‐expression ratio was approximately 88.1%, but it was rarely expressed in neurons (8.6%) and astrocytes (3.3%) (Figure 4E,F). To further validate above findings, we carried out the immunofluorescence assay of DBP within BV2 cells cultured in vitro, and found that DBP completely merged in BV2 cells (Figure 4G). Lastly, we detected the expression of DBP in the three types of hippocampal cells from the control and CP mice. Consistent with DEGs assay, DBP expression was selectively and significantly increased in microglia, not in neurons or astrocytes (Figure 4H). In addition, because previous report shows that ERK pathway was the downstream component of DBP,^[^ ^21^ ^]^ so we purified and detected the microglial ERK pathway, and found there was a significant increase in the ratio of p‐ERK/ERK in microglia (Figure S4, Supporting Information). These findings prove that DBP was mainly expressed in microglia and may mediate CP‐induced behavioral abnormality.

Increased DBP was reported to be related with spatial learning and susceptibility to kainate‐induced seizures.^[^ ^14^ ^]^ However, the effects of DBP on CP‐induced behavioral abnormality remain poorly resolved, so we asked whether microglial DBP mediates behavioral abnormality induced by CP. To examine this, we constructed adeno‐associated virus (AAV) with shRNA targeting DBP, and then delivered it into the hippocampus of Cxcr1‐iCre mice to knockdown DBP gene expression (Figure 4I). The microinjected site and silencing efficiency were identified by immunofluorescence and Western blotting assays, respectively (Figure S5, Supporting Information). As expected, we observed that DBP knockdown in microglia abolished the depression‐like behaviors induced by CP, such as reversing the decreased sucrose preference in SPT (Figure 4J), and decreasing the immobility time in TST and FST (Figure 4K,L). Additionally, similar effects were observed in the EPM and OFT tasks, that is, decreasing microglial DBP expression improved the anxiety‐like behaviors induced by CP, including enhanced open arm duration, distance and entries in the EPM test (Figure S6A‐E, Supporting Information), and increased central time, distance and crossings in the OFT (Figure S6F‐J, Supporting Information). Moreover, we evaluated spatial memory using Morris water maze, and detected that DBP knockdown in microglia shortened the latency from the fifth day during the learning stage (Figure 4M,N). During the probe trial test, selective knockdown of microglial DBP shortened the longer latency for the first crossing induced by CP, increased the crossings to the platform region, and prolonged the swimming time in the target quadrant, with no changes in swimming speed (Figure 4O–S). Together, these findings suggest that microglial DBP is required for depression‐like behaviors and memory deficits induced by CP.

### 2.5 Knockdown of Microglial DBP Reverses Synapse Loss and Neurogenesis Impairment Following CP

**Figure 5:**
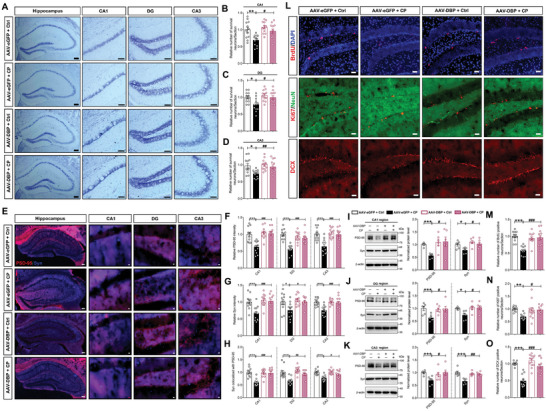
Knockdown of microglial DBP reverses synapse loss and neurogenesis impairment following chronic periodontitis. A‐D) Representative immunofluorescence images and statistical results of Nissl staining in the three sub‐regions of hippocampus from the Ctrl and CP mice treated with AAV‐eGFP or AAV‐DBP. The right is magnified staining images of hippocampal sub‐regions of CA1, DG, and CA3. Scale bar, 300 µm in left and 100 µm in enlarged images. E) Double‐staining of PSD‐95 (blue) and synaptophysin (green) in the hippocampus of Ctrl and CP mice treated with AAV‐eGFP or AAV‐DBP. The right is magnified staining images of hippocampal sub‐regions of CA1, DG, and CA3. Scale bar, 300 µm in left and 100 µm in enlarged images. F‐H) Statistical results of PSD‐95 intensity (F), synaptophysin intensity (G), and colocalized ratio between PSD‐95 and synaptophysin (H) in the hippocampal sub‐regions of CP mice relative to AAV‐eGFP + Ctrl mice. I‐K) Representative Western blots and quantification of PSD‐95 and synaptophysin expression in the CA1 (I), DG (J), and CA3 (K) sub‐regions of CP mice relative to AAV‐eGFP + Ctrl mice. L) Representative BrdU‐ and DAPI‐stained images, Ki67‐ and NeuN‐stained images, and DCX‐stained images of hippocampus from Ctrl and CP mice treated with AAV‐eGFP or AAV‐DBP. Scale bar, 100 µm. M‐O) Quantification of the relative number of BrdU positive (M), Ki67 positive (N), and DCX positive (O) neurons in hippocampus from Ctrl and CP mice treated with viral injection. All data are presented as mean ± SEM. Two‐way ANOVA for all. n = 12 per group for (B‐D, F‐H, M‐O), and n = 6 per group for (I‐K). *p < 0.05, **p < 0.01 and ***p < 0.001 versus AAV‐eGFP + Ctrl group. ^#^ *p* < 0.05, ^##^ *p* < 0.01 and ^###^ *p* < 0.001 versus AAV‐eGFP + CP group.

GO functional enrichment network of DEGs from the control and CP mice suggests that CP induced neuronal apoptosis and division and proliferation impairment of neuronal stem cells, and our results also validated this. Thus, we asked whether DBP is a critical factor in these processes. Based on our above findings, firstly, we examined the neuronal apoptosis by Nissl staining. As the results shown, DBP knockdown in microglia ameliorated the level of apoptosis induced by CP in the three hippocampal sub‐regions (**Figure** **5**A–D). Furthermore, we checked the synapse loss by synaptic markers in the DG, CA1 and CA3 regions. Consistent with the finding above, we found that selective knockdown of microglial DBP reversed the decreased staining of synaptophysin, PSD‐95 and the merge ratio of them in the three hippocampal sub‐regions (Figure 5E–H). Additionally, we further verified this using Western blotting and the results showed that, consistent with synaptic markers tests, decreasing the expression of DBP in microglia improved the CP‐induced downregulated expression of PSD‐95 and synaptophysin in the three hippocampal sub‐regions (Figure 5I–K). Finally, we detected the neurogenesis in the DG region, and observed that the CP‐induced neurogenesis impairment was recovered by selective knockdown of microglial DBP, which includes reversing the decreased numbers of BrdU and Ki67 positive cells and DCX positive cells in the DG region (Figure 5L–O). Taken together, these results reveal that microglial DBP plays an important role in synapse loss and neurogenesis impairment following CP.

### 2.6 CP Regulates Microglial Phenotype and Neuroinflammation Through DBP

**Figure 6:**
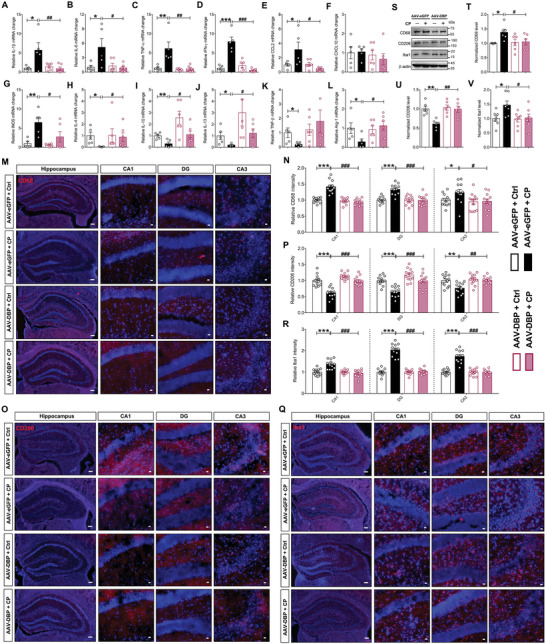
Chronic periodontitis regulates microglial phenotype and neuroinflammation through DBP. A‐L) Statistical analysis of mRNA changes of IL‐1β (A), IL‐6 (B), TNF‐α (C), IFR‐γ (D), CCL2 (E), CXCL10 (F), iNOS (G), IL‐4 (H), IL‐10 (I), IL‐13 (J), TNF‐β (K) and Arg‐1 (L) in the hippocampus tissues from the Ctrl and CP mice treated with AAV‐eGFP or AAV‐DBP. M, N) Representative immunofluorescence images and statistical results of CD68 in the hippocampus from the Ctrl and CP mice treated with AAV‐eGFP or AAV‐DBP. The right is magnified staining images of hippocampal sub‐regions of CA1, DG, and CA3. Scale bar, 300 µm in left and 100 µm in enlarged images. O, P) Representative immunofluorescence images and statistical results of CD206 in the hippocampus from the Ctrl and CP mice treated with AAV‐eGFP or AAV‐DBP. The right is magnified staining images of hippocampal sub‐regions of CA1, DG and CA3. Scale bar, 300 µm in left and 100 µm in enlarged images. Q, R) Representative immunofluorescence images and statistical results of Iba1 in the hippocampus from the Ctrl and CP mice treated with AAV‐eGFP or AAV‐DBP. The right is magnified staining images of hippocampal sub‐regions of CA1, DG and CA3. Scale bar, 300 µm in left and 100 µm in enlarged images. S‐V) Representative Western blots and quantification of CD68, CD206 and Iba1 in hippocampus from the Ctrl and CP mice treated with AAV‐eGFP or AAV‐DBP. All data are presented as mean ± SEM. Two‐way ANOVA for all. n = 12 per group for (A‐L, N, P, R), and n = 6 per group for (T‐V). *p < 0.05, **p < 0.01 and ***p < 0.001 versus AAV‐eGFP + Ctrl group. ^#^ *p* < 0.05, ^##^ *p* < 0.01 and ^###^ *p* < 0.001 versus AAV‐eGFP + CP group.

**Figure 7:**
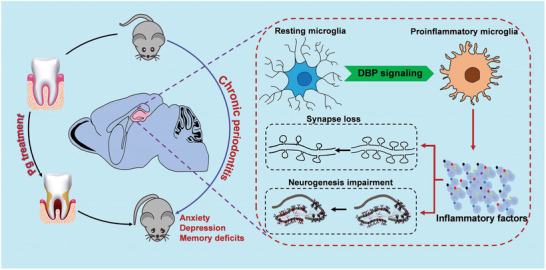
Schematic illustration of the mechanism of CP‐induced abnormality in emotional behaviors and memory deficits. Chronic periodontitis specifically increases the expression of DBP in the hippocampal microglia, subsequently induces the activation of resting microglia, resulting in the secretion of multiple inflammatory factors, and then mediates synapse loss and neurogenesis impairment in the hippocampus, and finally, facilitates the anxiety‐ and depression‐like behaviors and memory deficit in mice.

Previous study reports that DBP is involved in the progression of mesangial proliferative nephritis through regulating inflammatory cytokines expression,^[^ ^11^ ^]^ and our findings also showed that microglial DBP mediated synapse loss and neurogenesis impairment following CP. Further, we asked what effects DBP has directly on microglia in the hippocampus. In addition, there are increasing amounts of evidence showing that CP promotes microglial M1 polarization upon invasion of periodontal pathogen, and induces the secretion of inflammatory factors.^[^ ^22^ ^]^ Therefore, we wondered whether DBP mediates microglia activation and associated inflammation responses following CP. First, we tested the microglial M1/M2 associated markers and inflammatory factors. In accordance with previous studies,^[^ ^6^, ^23^ ^]^ we observed that there was a significant increase in microglial M1 markers and an obvious decrease in microglial M2 markers in the hippocampus of CP mice, and these alterations were reversed by knockdown of microglial DBP (**Figure** **6**A–L). Moreover, we further validated these findings by immunofluorescence assay with microglial M1/M2 polarization markers CD68 (M1) and CD206 (M2) in the hippocampus. Compared with the control mice, a significant increase in the number of CD68 staining positive cells was observed, and it was abolished by DBP knockdown in microglia in the CA1, DG, and CA3 regions (Figure 6M,N). Then, we also found that the number of CD206 staining positive cells was decreased in the hippocampus after CP, and this change was reversed by silencing DBP in microglia (Figure 6O,P). Next, the number and morphology of microglia was examined by Iba1 staining, and similar to CD68 staining, we observed that the number of Iba1 staining positive cells was significantly increased after CP, and this increase was abolished by transfection with AAV‐DBP in microglia (Figure 6Q,R). Additionally, we also found that morphology of microglia was changed from resting state into amoeba‐shaped state, and this phenomenon was reversed following DBP knockdown in microglia (Figure S7, Supporting Information). Lastly, above findings were further verified by Western blotting. As the results showed, silencing DBP in microglia reversed the increased CD68 and Iba1 expression, and the decreased CD206 expression in the hippocampus induced by CP (Figure 6S–V). Collectively, these results demonstrate that CP preferentially increased the expression of DBP in hippocampal microglia, which was followed by activation of resting microglia and increased secretion of multiple inflammatory factors, and then resulted in synapse loss and neurogenesis impairment in the hippocampus, subsequently mediating the anxiety‐ and depression‐like behaviors and memory deficit in mice (**Figure** **7**).

## 3 Discussion

In the present study, we demonstrated that CP could induce behavioral abnormality such as anxiety‐ and depression‐like behaviors and spatial learning deficits, accompanied by hippocampal synapse loss and neurogenesis impairment. DBP was screened as the most prominently upregulated gene by RNA microarray, and it was shown to be mainly expressed in microglia. Knockdown of microglial DBP ameliorated the CP‐mediated behavioral abnormality, hippocampal synapse loss and neurogenesis impairment, neuroinflammation and microglial activation, and neuroinflammation.

Periodontal disease and dental caries are the two main dental diseases that are relevant to people with mental illness.^[^ ^24^ ^]^ Dental caries usually does not cause mental illness, while there are increasing amounts of evidence showing that periodontal patients have a significantly higher total depression score than normal controls.^[^ ^25^ ^]^ Additionally, more research results suggest that CP could induce an impairment of learning memory.^[^ ^5^, ^26^ ^]^ We also verified and found that mice subjected to CP exhibited spatial learning memory deficit in Morris water maze and Barnes maze tests. Consistent with this, our fEPSP recordings from hippocampal slices showed that CP caused a lesion of SC‐CA1 LTP. In contrast to the CP‐induced deficits in spatial memory, there was no significant difference in cue and contextual fear conditioning induced learning memory between Ctrl and CP groups. Why did mice show different behavioral results in terms of memory? According to previous studies, a possible explanation for this might be like that. Contextual fear memory depends on hippocampus and amygdala, while cued associative fear memory depends only on amygdala but not the hippocampus. Thus, the hippocampus, particularly dorsal hippocampus, is more important for spatial memory.^[^ ^27^ ^]^

Although the effects of CP on depression‐like behaviors and spatial learning memory were confirmed, the possible mechanisms coupling CP with behavioral abnormality remain poorly understood. The recent study revealed that having fewer teeth is associated with more serious atrophy of left hippocampus in patients with mild periodontitis, whereas having more teeth is associated with a faster rate of atrophy in those with severe periodontitis.^[^ ^4^ ^]^ This suggests that CP may promotively induce apoptosis of the hippocampal neurons. To examine this hypothesis, we detected the morphology of the cells in hippocampus of the CP group by Nissl staining. Consistent with previous findings, we found that neurons in the hippocampus of the CP group were loosely arranged and the number of Nissl bodies was also reduced. Furthermore, in several studies, CP has been shown to regulate neurogenesis in the hippocampus.^[^ ^4^, ^7^ ^]^ We detected this by high‐throughput sequencing and immunofluorescence experiments, and the results showed that CP impeded the neuronal stem cell division and proliferation. However, little is known yet about the underlying mechanisms. Accumulated studies suggest that microglia‐mediated neuroinflammation is involved in hippocampal neuron apoptosis and neurogenesis damage induced by CP.^[^ ^28^ ^]^ But other studies showed that both astrocytes and microglia were activated in the hippocampus through TLR4/NF‐κB signaling pathway.^[^ ^29^ ^]^ Certainly, another report suggested that Pg induces depression via downregulating p75NTR‐mediated BDNF maturation in astrocytes.^[^ ^30^ ^]^ Thus, to verify which one contributes to the alterations of hippocampal morphology induced by CP, we tested the level of pro‐inflammatory cytokines by RT‐PCR and found that CP promoted neuroinflammatory responses in the hippocampus, and the number of microglia was significantly increased, while no obvious change in the number of astrocyte was observed. Moreover, selectively knockdown microglial DBP promoted the polarization of microglia from M1 to M2 and inhibited the expression of inflammatory cytokines, as well as attenuated synapse loss and neurogenesis impairment induced by CP. These findings further demonstrated that microglia‐mediated neuroinflammation plays a critical role in the alterations of hippocampal morphology caused by CP.

One recent study documented that DBP could effectively inhibit the proliferation of primary rat mesangial cells through G1 phase arrest,^[^ ^31^ ^]^ and our results showed that CP‐induced microglial DBP expression mediated synapse loss and neurogenesis impairment in the hippocampus. These findings raise a question about the possible mechanisms underlying the effects of microglial DBP on regulating neuronal growth and development in the hippocampus. Another problem before solving this one is that the cell type specificity of DBP in hippocampus is still unclear. Here, we found that DBP was mainly located in microglia, not in neurons or astrocytes in hippocampal tissue, and the same phenomenon was observed in experiments at the cell level. Next, we examined the expression of DBP in the above three types of cells in the CP mice. Consistently, DBP expression was significantly increased in microglia in vitro, not in neurons or astrocytes, suggesting that microglial DBP mediates hippocampal synapse loss and impairment of neurogenesis after CP. As expected, we also found that there is microglial activation and neuroinflammation in hippocampus following CP, which could be abolished by DBP knockdown in the microglia. These results support that DBP may induce the activation of resting microglia, resulting in neuroinflammation, which then mediates synapse loss and neurogenesis impairment in the hippocampus.

Although our findings demonstrated that DBP may be pivotal in the CP‐induced microglia activation and the associated neuroinflammation, the underlying mechanisms of DBP coupling CP with microglial activation are unclear. Previous study revealed that two‐fold higher level of activated MAP kinase (MAPKs) in hippocampal dendrites was detected following rAAV‐DBP injection,^[^ ^16^ ^]^ and emerging evidence also showed that MAPKs signaling pathway plays a critical role in the microglial activation in hippocampus after chronic stress.^[^ ^32^ ^]^ Here, we purified and detected the microglial ERK pathway, and found there was a significant increase in the ratio of p‐ERK/ERK in microglia. However, the precise mechanism of action of DBP on microglial ERK pathway activation following CP is still unknown. Additionally, DBP is reported to be a circadian protein, whose expression is almost undetectable in the morning and peaks at approximately 8:00 PM in rat liver.^[^ ^33^ ^]^ We wonder if a similar DBP rhythm phenomenon would occur in the brain, especially in the hippocampus. Furthermore, mice were administrated with Pg suspension via oral gavage, and the distance from CEJ to ABC in the maxilla on the buccal and palatal surfaces, and inflammatory cell infiltration in three groups of mice was examined, coincidentally, the alterations of trend of distance and inflammatory cell infiltration kept pace with the levels of anxiety‐ and depression‐like behaviors in mice after CP, these results suggested that CP‐induced effects on the brain may depend on degree of periodontal disease inflicted, although no direct analysis between brain function and periodontal disease. Besides, our results revealed that chronic periodontitis mice are prone to depression and memory deficit, thus we are curious about whether chronic stress increases the susceptibility of periodontitis, this question is interesting but remain to be verified

In conclusion, the results of this study showed an important effect of microglial DBP signaling on CP‐induced behavioral abnormality in mice. Our findings indicate that DBP plays a critical role in microglia activation and neuroinflammation, followed by neuronal apoptosis and neurogenesis impairment in the hippocampus, and this disturbance of synaptic signaling transmission causes depression‐like behaviors and spatial learning memory deficit. Therefore, our findings provide direct evidence for the role of microglial DBP signaling in the CP‐induced emotional and cognitive deficit, and our results also indicate that microglia activation through the DBP signaling may be a promising target for the treatment of CP‐related emotion and learning memory disorders.

## 4 Experimental Section

### Mice

Male C57BL/6J mice were purchased from the Laboratory Animal Center of Hangzhou Medical College (Hangzhou, China; certificate number: SCXK2019‐0002). Cx3cr1‐iCre mice were obtained from Jie‐Yan Zheng (Guangdong Provincial Key Laboratory of Brain Function and Disease, Zhongshan School of Medicine, Sun Yat‐sen University). Mice were caged in groups of 4–5 mice and maintained in a 12‐h light/dark cycle, in stable conditions of temperature at 24 ± 2 °C and relative humidity of 50% ± 5%, with free access to food and water. Mice (4–5 weeks, 20 ± 2 g) were used for experiments and were habituated to the environment for at least 1 week before being subjected to experiments. All the experiments were conducted under the approval of the Institutional Animal Care and Use Committee of Fujian Medical University.

### Cell Culture

Mouse BV2 cell line was purchased from the Jennio Biotech Co. Ltd (Guangzhou, China), and cultured in Dulbecco's Modified Eagle Medium supplemented with 10% fetal bovine serum and 1% penicillin/streptomycin in a humidified incubator of 5% CO_2_ at 37 °C.

### Chronic Periodontitis Model

The chronic periodontitis (CP) model building was performed as described previously.^[^ ^6^ ^]^ *Porphyromonas gingivalis* (Pg) was purchased from Shanghai Microbiological Culture Collection Center (Shanghai, China), and Pg was suspended at a final concentration of 1.5×10^8^ CFU in 0.1 mL PBS with 2% carboxymethyl cellulose (CMC). Next, all mice were randomly divided into two groups: the control (Ctrl) group and the chronic periodontitis (CP) group. For the CP group, the Pg suspension was supplied to each mouse by oral gavage four times per day for one month, and the drinking water was changed to 10% sucrose solution every other day. While for the Ctrl group, mice were subjected to 0.1 mL PBS with 2% CMC without Pg and accessible to drinking water only. For the two groups, food was adequate and free to access during the entire process, and the body weight of each mouse was monitored every two days.

### Sucrose Preference Test

Sucrose preference test (SPT) was used for assessing anhedonia in mice, and the procedure was conducted as previously described.^[^ ^34^ ^]^ Briefly, mice were singly housed and given one bottle of 1% sucrose solution and one bottle of drinking water for 24 h. The positions of the two bottles were switched for another 24 h adaption to avoid a side bias, and all mice have free access to chow. Then, mice were deprived of both food and water prior to test. After 24 h deprivation, mice were subjected to a two‐bottle choice test for 24 h, with two bottles containing 1% sucrose solution and drinking water respectively. During the test, the position of two bottles was switched after the first 12 h and food was accessible. Tips of two bottles were placed adjacently and each bottle was weighed before and after the test. The anhedonia was measured by sucrose preference ratio, presented as a percentage of sucrose consumption relative to the total consumption of sucrose solution and drinking water.

### Tail Suspension Test

Tail suspension test (TST) was used to assess depression or despair in rodents and the procedure was carried out as previously described with slight modifications.^[^ ^35^ ^]^ The test apparatus was a case made of white acrylic sheets, with one wall open for recording. During the test, a tape was fixed 1 cm from the tip of mouse tail, and each mouse was suspended 20 cm above the floor through the tape for 6‐min test. When mice performed no body movement or hugged passively for 3 s, this behavior was considered as immobility. The resultant behavior during the test was recorded by a camera linked to computer and the time mice spent immobile was analyzed. Immediately after the test, the tape affixed to its tail was removed and the mouse was transferred back to home cage.

### Forced Swimming Test

Forced swimming test (FST) was also used to examine depression‐like behaviors, and was performed based on previously described procedure.^[^ ^36^ ^]^ In detail, a transparent cylinder at 15 cm in diameter and 35 cm in height was used as the test apparatus. Before the test, the cylinder was filled with water to a depth of 20 cm and the water temperature was kept at 23–25 °C. Then, each mouse was gently placed in the cylinder for 6 min, and had the video recorded. The immobility was defined as the mice stopped struggling for 3 s, and the immobility time during the final 5 min interval was analyzed. After the test, each mouse was returned to its home cage and stayed in a warm place until the fur became dry.

### Open Field Test

Open field test (OFT) was utilized to evaluate the baseline level of motor activity and anxiety‐like behaviors in rodents, and OFT was performed as previously described with slight modifications.^[^ ^37^ ^]^ Briefly, a plastic open‐field apparatus (50×50×40 cm) was used in this test and was cleaned after each trial to avoid clues of odour. The open field was divided into a central 30×30 cm center zone and a peripheral zone. Each mouse was gently placed in the center zone of field and was allowed to freely explore for 5 min under mild white light. During the test, the motion trail, the total travel distance and the time spent in the center zone or in the peripheral zone were monitored and analyzed by a video camera hanging above the apparatus.

### Elevated Plus Maze Test

Elevated plus maze (EPM) test was used to measure anxiety‐like behaviors and was performed as previously described with slight modifications.^[^ ^38^ ^]^ The EPM apparatus consisted of four arms (5 cm in width and 30 cm in length for each), which were perpendicular to each other and formed a cruciform. While two opposite arms, enclosed with walls of 15 cm high, were defined as the closed arms, the other two arms were defined as the open arms. Before test, the apparatus was elevated 40 cm above the floor. Each mouse was gently placed in the crossing center area of plus maze and faced one of open arms, and was allowed to explore for 5 min under dim light. An overhead video camera was utilized to record the open‐arm entries, open‐arm distance and the time spent in the open arms. The EPM apparatus was thoroughly cleaned with ethanol after each trial.

### Morris Water Maze Test

Morris water maze (MWM) test was used to assess the spatial learning ability and memory in rodents, and the MWM test was conducted based on previously described procedure with minor modifications.^[^ ^39^ ^]^ The maze was 150 cm in diameter and 50 cm in height, and was filled with water to the height of 35 cm. The water temperature was kept at 20–22 °C, and the apparatus was surrounded by curtains. The maze was divided into four equal quadrants, one of which was equipped with a submerged invisible platform 1.5 cm below the water surface, measuring 10 cm in diameter. The position of the platform was permanent throughout the course of the MWM test. Four diverse geometries were stuck to the pool side of four quadrants respectively, which was used to help mice to recognize the position of escape platform and to better form spatial memory. At the training phase, each mouse was gently placed into water in one of the four quadrants, facing the pool wall, and was allowed to freely explore for 1 min. If the mouse could not find the escape platform within 1 min, the mouse was guided to the platform and stayed for 15 s to form spatial learning and memory. However, when the mouse found the platform within 1 min, the trajectory and latency to escape were recorded and the mouse also had a 15 s stay on the platform for learning and memory. Each mouse was trained for 6 consecutive days with 4 trials per day from the four quadrants respectively, and a time interval of more than 15 min was needed by one mouse between two trials. The spatial probe trial was performed on day 8. Before testing, the platform was withdrawn, and the mouse was placed in the opposite quadrant. The motion trail, total travel distance, the time spent in the quadrant of escape platform, the number of platform area crossings and the latency of the first platform area crossing, were tracked and recorded for 1 min by the overhead video camera.

### Barnes Maze Test

Barnes maze test was also an assessment for spatial learning memory, and it was performed as previously described.^[^ ^40^ ^]^ In detail, the test apparatus was an open circular platform measuring 120 cm in diameter and there were 20 equal holes at 5 cm in diameter along the perimeter of the platform with identical gap. One of the holes was considered as the target hole, and underneath the target hole equipped with a black opaque acrylic box called the target chamber, which mice could escape into. Around the apparatus decorated four diverse geometries to facilitate spatial orientation. The position of the target hole was permanent throughout the test.

Before training, mice were placed in the middle of platform with a black cylindrical start chamber covered for 10 s. Aversive noise at 80 dB and bright light were used to motivate mice to enter the target hole. After that, the start chamber was removed and mice were guided into the target chamber. Once mouse entered the chamber, the noise was turned off and two minutes were given for adaptation in the chamber. On training phase, the training was started by placing a mouse into the same start chamber for 10 s with noise turned on. Then, the start chamber was lifted and the activity of the mouse was observed for 3 min by an overhead camera. If mice found and escaped into the target chamber within limited time, the noise was immediately stopped and mice were remained in the chamber for 1 min. However, if mouse was unable to find the target hole after 3 min, they were guided into the chamber and also remained for 1 min. During this phase, each mouse was trained 4 times per day for 4 consecutive days, with a 15 min interval between two adjacent trainings, and the latency of escape was recorded in each trial during this process. The short‐term spatial memory was then tested on day 5, and the target chamber was removed before test. In the same way, mouse was placed in the start chamber and the test began after the start chamber was lifted. The motion trajectory, total travel distance, the number of and time spent in exploring the target hole, and the latency of the first target hole exploration, were analyzed for 2 min to monitor animals’ spatial learning and memory. The entire maze and the target chamber were fully cleaned with ethanol and dried after each trial to avoid odour cues from mice.

### Cue Fear Conditioning

Cue fear conditioning experiment was conducted over 3 days, day 1 (habituation), day 2 (fear conditioning) and day 3 (testing). Mice were exposed to the chamber for 5 min on day 1. On day 2, after 3 min of habituation in the chamber (Context A), mice were presented with 5 presentations of a tone conditioned stimulus (CS, 65 dB, and 29 s), each co‐terminated with a 0.75 mA foot shock lasting 1 s as an unconditioned stimulus (US), and the intertrial intervals between CS–US of 30 s. Mice were removed from the conditioning chambers immediately after the session. On day 3, the mice were placed in another chamber (Context B) and presented with five CS tone presentations (65 dB, and 29 s), and observed for 5 min. The percentage of freezing time was measured by the ANY‐maze software (Stoelting Co., IL, USA).

### Contextual Fear Conditioning

The procedure of contextual fear conditioning was similar to that of the cue fear conditioning with some modifications. Briefly, on day 1 (habituation day), mice were exposed to the chamber for 5 min. On day 2 (fear conditioning day), mice were given five 1‐s foot shocks at 0.75 mA with the 90 s‐ intertrial intervals. Then, the mice were removed from the chambers immediately after the session. On day 3 (testing day), the mice were placed in the same chambers and observed for 5 min. The percentage of freezing time was recorded by ANY‐maze software (Stoelting Co., IL, USA).

### Slice Preparation

Mouse was anesthetized with isoflurane (2%) and transcardially perfused with cutting solution (4 °C) containing (in mM): 135 N‐methyl‐D‐glucamine, 1 KCl, 1.2 KH_2_PO_4_, 20 choline‐HCO_3_, 11 glucose, 0.5 CaCl_2_, 1.5 MgCl_2_, pH adjusted to 7.4 with HCl and saturated with 95% O_2_–5% CO_2_. The brain was dissociated after quick cutting and decapitation, and was immediately immersed into ice‐cold oxygenated cutting solution, and then the brain was cut into coronal bilateral slices (300 µm in thickness) containing hippocampus by a vibratome (VT 1200S, Leica, Wetzlar, Germany) in aCSF (in mM: 119.0 NaCl, 1.3 MgSO_4_, 3.5 KCl, 1.0 NaH_2_PO_4_, 26.2 NaHCO_3_, 2.5 CaCl_2_ and 11.0 glucose). Next, the slices were transferred to the holding chamber with aCSF to incubate at 34 °C for 30 min, and then the slices were incubated at 28 °C in aCSF, which was continuously perfused with oxygen.

For the collection of samples, after cutting, the slices were transferred to a glass dish for further sectioning the hippocampal tissue under the microscope. Both dorsal and ventral hippocampus region were isolated with syringe needles, and were collected into EP tube for Western blotting or RT‐PCR assays.

### LTP Recording

LTP induction and recording was performed as described previously.^[^ ^41^ ^]^ In detail, conditioning stimulation consisted of 360 pulses at 2 Hz was paired with continuous postsynaptic depolarization (180 s) to 0 mV. To suppress excessive polysynaptic activity, picrotoxin (50 µM) was added in the recording bath, and the concentration of divalent cations was elevated to 4 mM Ca^2+^ and 4 mM Mg^2+^ to reduce recruitment of polysynaptic responses. A test pulse was delivered at 0.05 Hz to monitor baseline amplitude for 10 min before and for 25–35 min following paired stimulation. To calculate LTP, the EPSC amplitude was normalized to the mean baseline amplitude during 10 min baseline. Potentiation was defined as the mean normalized EPSC amplitude 25–40 min after paired stimulation. Data were acquired in an interleaved manner for LTP comparisons between different groups.

### Stereotaxic Injections

Mice were anesthetized with 2% isoflurane, and then fixed on a stereotaxic apparatus (RWD Life Science, China). Furs on the skull were shaved and erythromycin was applied to both eyes to avoid keratitis. Then, a straight incision from the middle of ears to the interpupillary line was made, and the head was leveled horizontally with the bregma as the coordinate origin. A burr hole in the skull above the hippocampus was made by a drill. The virus of AAV‐eGFP or AAV‐DBP (titers: 5.00×10^8^ VG/mL, purchased from Zolgene Biotechnology Co., Ltd, Fuzhou, China) was microinjected into the hippocampus region of the left and right hemisphere, of which the injection position was 2.7 mm posterior to the bregma, ± 2.0 mm lateral and 2.0 mm blow the level of bregma, at a rate of 0.05 µL per min with a total volume of 0.5 µL per side using a microsyringe (1 µl) attached to the apparatus. The needle was left in place for additional 5 min to ensure adequate diffusion and then was withdrawn slowly. After the injection of both sides was finished, the skin overlying the cranium was sutured. Mice were then kept in a warm blanket to completely recover from anesthesia before returning to the home cages.

### Magnetic Bead Sorting‐Based Isolation of Microglia, Astrocytes, and Neurons

This assay was performed as described previously with minor modification.^[^ ^42^ ^]^ After hippocampal tissue collection, according to the manufacturer's instruction (Miltenyi Biotec cell dissociation kit #130‐107‐677), the hippocampal tissue was clean with pre‐cooling D‐PBS and then incubated with enzyme mixture at 37 °C for 30 min to obtain dissociated cells. D‐PBS was used to re‐suspend cell precipitation and appropriate volume of debris removal solution was added to evenly mix. D‐PBS (4 mL) was then added to stop the reaction, and the mixture was centrifuged at 4 °C, 3000 g for 10 min. The suspension was divided into 3 layers. The top two layers were removed. The bottom layer was mixed with D‐PBS and centrifuged at 4 °C, 1000 g for 10 min. The supernatant was removed and an appropriate volume of erythrocyte lysate was added. Cells were incubated at 4 °C for 10 min. 10 mL D‐PBS was added to stop the reaction, and the mixture was centrifuged at 4 °C, 300 g for 10 min, to remove the supernatant. Magnetic bead antibody for microglial membrane surface marker was added into cell suspension and incubated at 4 °C for 15 min under dark conditions. The cells were eluted into the centrifuge tube and centrifuged at 4 °C and 300 g for 5 min to obtain microglial precipitation. Magnetic bead antibody for astroglial membrane surface marker was then added to obtain astrocyte precipitation following the above procedure. Neurons were obtained by adding magnetic bead antibody that depleted all non‐neuronal cells to acquire highly purified neurons from mouse brain tissue.

### Western Blotting

Samples were homogenized and sonicated in radioimmunoprecipitation assay (RIPA) buffer (50 mM Tris‐HCl, pH 7.4, 1% NP‐40, 0.5% Na‐deoxycholate, 0.1% SDS, 150 mM NaCl, 2 mM EDTA and 50 mM NaF; 10 µL mg^−1^) supplemented with protease inhibitors PMSF (1%) and PI (1%). The lysates were then centrifuged at 12 000 g for 15 min at 4 °C, and the supernatant was collected, and the concentration of supernatant was measured by Coomassie blue protein‐binding assay (Nanjing Jiancheng Institute of Biological Engineering, Nanjing, China) with bovine serum albumin (BSA) as a standard. Clarified samples were boiled at 95 °C for 5 min with SDS‐loading buffer, and then the final protein samples were loaded on 10% SDS‐polyacrylamide gel electrophotesis (PAGE) and transferred to polyvinylidene difluoride (PVDF) membrane. After that, bands at the location of target proteins were incubated in 5% BSA (w/v, in Tris‐buffered saline, pH 7.4 and with 0.5% Tween‐20, TBST) for 1.5 h at room temperature before incubated with primary antibody solution (1% BSA and primary antibodies in TBST) overnight at 4 °C. The bands were washed in TBST three times for 20 min at room temperature and incubated with horseradish peroxidase (HRP)‐conjugated secondary antibody (1% BSA and secondary antibodies in TBST) for another 2 h at room temperature. After washing three times at room temperature for 10 min with TBST, the bands were reacted with enhanced chemiluminescence substrate (Super Signal West Pico; Pierce Chemical Co., Rockford, IL) for about 2 min or more time, and captured into the Micro Chemi (DNR Bio‐imaging systems, Jerusalem, Israel). The optical densities of the immunoblots were measured by NIH ImageJ software. All the results were presented as the percentage of control following normalization. The Information of antibodies used in Western blotting assays was described in the Table S1 (Supporting Information).

### Immunohistochemistry

For tissue preparation before immunofluorescence assay, mice were anesthetized with isoflurane (2%) and transcardially perfused with saline followed by 4% paraformaldehyde (PFA). The brain tissue was removed and fixed in PFA overnight at 4 °C, then dehydrated in 15% and 30% sucrose solution dissolved in phosphate buffer saline (PBS) at 4 °C for one day respectively. Then the brains were quickly embedded with O.C.T. compound and frozen to −20 °C.

Coronal sections at the thickness of 30 µm were generated by using freeze‐sectioning method on a freezing microtome (Leica CM19000). The intact sections were collected into a 24‐well plate and rinsed three times for 10 min in PBS. After incubating in blocking buffer (3% BSA solution 950 µl, 10% Triton X‐100 30 µl and donkey serum 20 µL) for 1 h at room temperature, the sections were again washed with PBS for three times, and then sections were incubated with primary antibody, which was dissolved in PBST at final using concentration, overnight at 4 °C. After washing in PBST three times for 10 min, the sections were labeled by incubating with fluorescent‐dye‐conjugated corresponding secondary antibody in a shaker at 80 g, 37 °C and away from light. The slices were washed three times for 10 min with PBS in a dark room and brain sections containing hippocampus regions were carefully loaded onto clean slides for further imaging. A drop of 30% glycerin was added to the brain sections to prevent sections from drying out and cracking. Images of hippocampus regions were acquired using a confocal laser scanning microscope. These operations were also performed away from light. The Information of antibodies used in immunohistochemistry assays was described in the Table S1 (Supporting Information).

### Measurement of Alveolar Bone Loss

Alveolar bone loss was analyzed as previously reported with minor modification.^[^ ^43^ ^]^ Briefly, the maxilla was dissected and defleshed after 10 min in boiling water, immersed overnight in 3% hydrogen peroxide, and stained with 1% methylene blue to visualize the cement enamel junction (CEJ). The distance from the CEJ to the alveolar bone crest (ABC) was measured at the palatal and buccal sides of the maxillae.

### H&E Staining

This procedure was performed following previous protocol.^[^ ^44^ ^]^ In brief, mouse maxillae were immersed in 4% paraformaldehyde solution for 24 h and decalcified in 10% ethylenediamine tetra acetic acid (EDTA) solution at 4 °C for four weeks. Then, the maxillae were subjected to ethanol gradient dehydration (70%, 85%, 95% ethanol for 40 min respectively) and embedded in paraffin with the buccal side of the tooth facing the bottom of the micromodel and the long axis of the tooth parallel to the short side of the cassette. Next, the sagittal sample sectioning to a thickness of 5 µm was performed, and then the sections were dewaxed in xylene, rehydrated by decreasing concentrations of ethanol, and washed in PBS. Lastly, the sections were stained with hematoxylin and eosin, and then dehydrated by increasing concentrations of ethanol and xylene, and digital images were acquired under an ECLIPSE Hi‐U microscope (Nikon, Tokyo, Japan).

### Nissl Staining

Nissl staining assay was performed as described previously.^[^ ^45^ ^]^ In detail, the O.C.T. compound embedded brains were cut at the thickness of 30 µm by freezing microtome (Leica CM19000). The slices were washed in PBS three times for 30 min at room temperature, and then incubated in 3% tritonX‐100 solution for 2 h. Next, the slices were incubated in toluidine blue (0.04%) at 37 °C for 10 min, washed in PBS for three times, and then rinsed with ethanol (95%) for 5 min and two times, followed by dehydrated in ethanol for 5 min. Lastly, the slices were transparent with xylene for 10 min, then sealed with neutral gum, and the staining of hippocampus regions was acquired using a microscope.

### Real‐Time Quantative PCR

Tissue samples of hippocampus from vibration sectioning were homogenized in 1 mL per 100 mg Trizol (Takara, Kyoto, Japan) for 30 min at room temperature, and then centrifuged at 12 000 g, 4 °C for 10 min, and the supernant was transferred to a new EP tube and added chloroform by one‐fifth Trizol volume. After extraction, the chloroform supernatant was collected and added equal volume of isopropanol for 10 min, followed by washing the precipitation with 75% ethanol solution. Lastly, the ethanol was removed by centrifugation at 7500 g, 4 °C, for 5 min and the RNA precipitation was naturally dried in a fume hood. RNase‐free dH_2_O was added to dissolve the RNA precipitation, and the concentration and purity of RNA were determined by a spectrophotometer. The samples with RNA purity at OD_260_/OD_280_ = 1.8‐2.0 were picked for reverse transcription. The reverse transcription reaction system (Yeasen Biotechnology, Shanghai, China) was as follows: 4 µl TransScript RT/RI Enzyme Mix, 1 µl gDNA Remover, total RNA (1 µg) and RNase‐free dH_2_O to a total volume of 20 µl. The synthesis of cDNA was performed in a program of 15 min at 42 °C and 5 sec at 85 °C.

The resulting cDNA was then used for qPCR in a 96‐well plate. Each sample was loaded for 3 wells and the reaction system in each well consisted of 5 µL 2× SYBR Premix Ex Tap II, 0.2 µL PCR Forward Primer, 0.2 µL PCR Reverse Primer, 0.2 µL cDNA and RNase‐free dH_2_O to a total volume of 10 µL. The RT‐qPCR conditions included 30 s at 95 °C and 40 repetitions of a cycle of 5 s at 95 °C paired with 3 s at 60 °C. For the analysis of the gene expressed in hippocampus, results were presented as linearized Ct‐value normalized to GAPDH and the ΔΔCt method was used to obtain the fold change of mRNA expression. The primer sequences used in this study were described in the Table S2 (Supporting Information).

### Statistical Analysis

All data were analyzed with GraphPad Prism 9 and SPSS 18.0 software (SPSS Inc, Chicago, IL, USA), and data were analyzed with independent‐samples two‐tailed t‐test, one‐way analysis of variance (ANOVA) or two‐way ANOVA as appropriate. All statistical results and tests used are included in the figure legends. Data were expressed as means ± S.E.M, and p value <0.05 was considered statistically significant.

## Conflict of Interest

The authors declare no conflict of interest.

## Author Contributions

T.C., D.T., S.‐Y.W., and Y.P. contributed equally to this work. T.C. performed the main part of the molecular biological experiments and wrote the manuscript. D.T. performed the behavioral tests and analyzed the data. S.‐Y.W. and Y.P. performed the immunofluorescence experiments. Z.‐X.X. performed the whole‐cell patch‐clamp recordings and analyzed the data. W.‐K.C. and S.‐W.Y. performed the stereotaxic surgery and microinjection. Q.‐Q.Z. cultured BV2 cells. Y.‐L.Z. collected the samples. L.Z. feed and took care of the animals. N.L. and Y.‐X.L. helped in reviewing and editing the manuscript. Z.‐C.S. and Z.‐M.L. supervised the project, designed the experiments and supported funding acquisition.

## Supporting information



Supporting Information

## Data Availability

The data that support the findings of this study are available from the corresponding author upon reasonable request.
