# Supplementary material for: Microglial DBP Signaling Mediates Behavioral Abnormality Induced by Chronic Periodontitis in Mice
Source: Adv Sci (Weinh). 2024 Oct 21;11(46):2406269. doi: 10.1002/advs.202406269 (PMC11633467; doi:10.1002/advs.202406269)
Supplement: Supplementary file 1 — Supporting Information [file ADVS-11-2406269-s001.docx]

**Supporting Information for**

**Microglial DBP signaling mediates behavioral abnormality induced by chronic periodontitis in mice**

Ting Cao, Dan Tian, Si-Ying Wang, Yue Pan, Zhi-Xuan Xia, Wei-Kai Chen, Shao-Wei Yang, Qing-Quan Zeng, Yue-Ling Zhao, Ling Zheng, Ning Li, Zhong-Meng Lai, Yi-Xiao Luo, Zu-Cheng Shen

**Supplementary Figures**

**
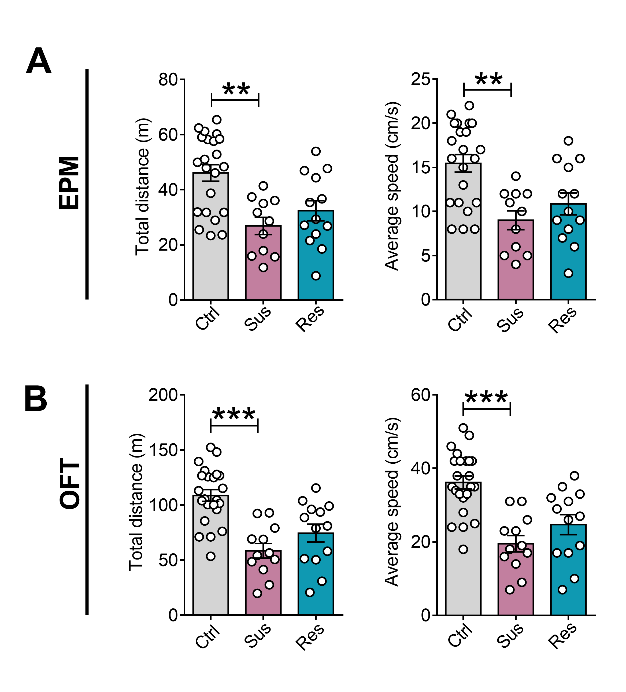
**

**Figure S1.** Changes in mice motor ability after chronic periodontitis. A-B) The total distance and average speed of Ctrl, Sus, and Res mice in the EPM (A) and OFT (B). n = 25 (Ctrl), 12 (Sus) and 14 (Res) in each group, respectively. All data are presented as the mean ± SEM. **p < 0.01 and ***p < 0.001 vs. Ctrl group using two-way ANOVA.


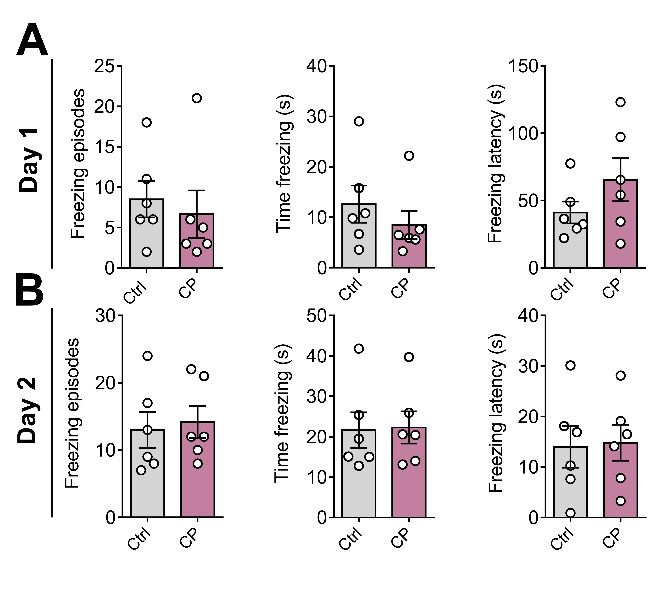


**Figure S2.** Baseline behaviors of Ctrl and CP mice in cue FC test. A-B) The freezing episodes, total freezing time duration and freezing latency of Ctrl and CP mice in cue FC test on Day 1 (A) and Day 2 (B), respectively. n = 6 per group. All data are presented as the mean ± SEM. Student’s t-test was used.


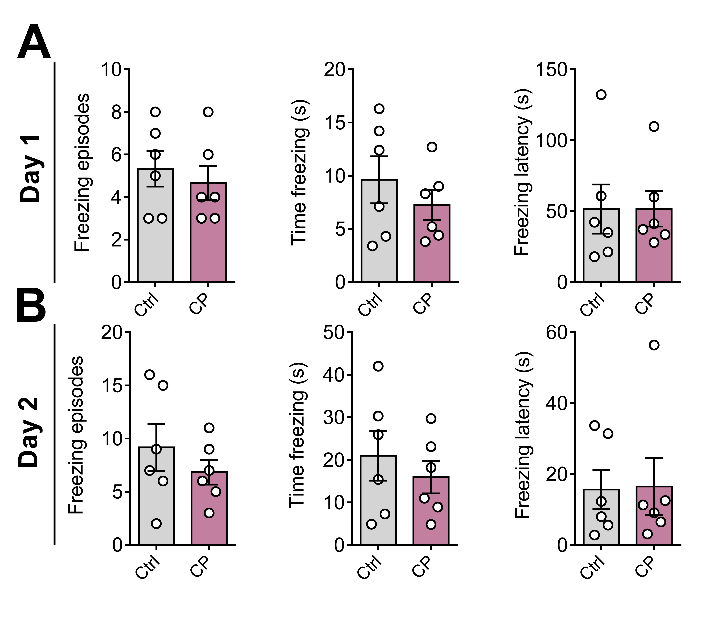


**Figure S3.** Baseline behaviors of Ctrl and CP mice in contextual FC test. A-B) The freezing episodes, total freezing time duration and freezing latency of Ctrl and CP mice in contextual FC test on Day 1 (A) and Day 2 (B), respectively. n = 6 per group. All data are presented as the mean ± SEM. Student’s t-test was used.


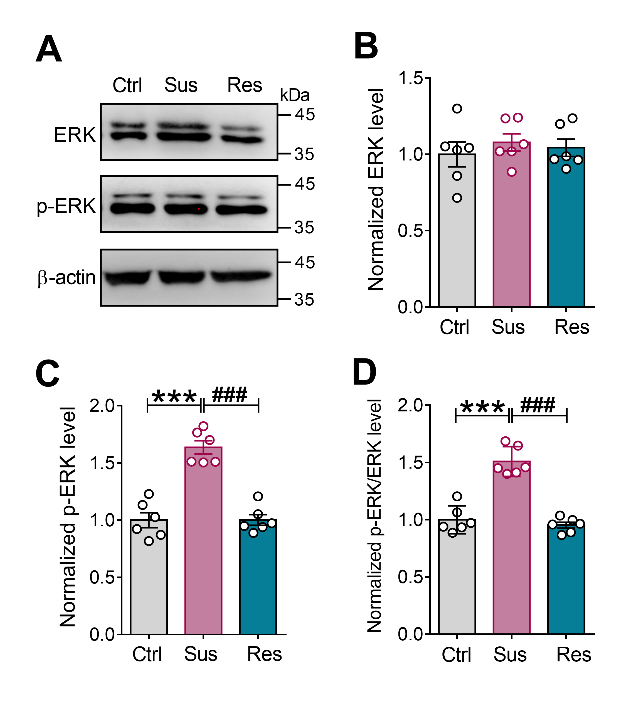


**Figure S4.** Effects of chronic periodontitis on the ERK signaling in the hippocampus. A) Representative Western blots of ERK and p-ERK expression in hippocampus of Ctrl, Sus, and Res mice. B-D) Statistical results of normalized ERK (B), p-ERK (C) and p-ERK/ERK (D) levels in the hippocampus of Ctrl, Sus, and Res mice. n = 6 in each group. All data are presented as the mean ± SEM. ***p < 0.001 vs. Ctrl group, ^###^p < 0.001 vs. Sus group using two-way ANOVA.

**
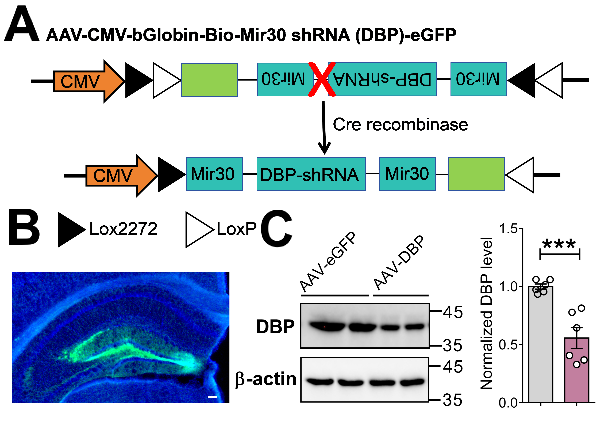
**

**Figure S5.** The structure of AAV-DBP and verification of virus injection. A) Schematic representation of construct of AAV-DBP. B) The immunofluorescent identification of coronal brain sections containing the hippocampus region. Scale bars: 100 μm. C) The representative Western blots and quantification of DBP expression in the hippocampus from AAV-eGFP and AAV-DBP groups. n = 6 per group. All data are presented as the mean ± SEM. ***p < 0.001 vs. AAV-eGFP group using Student’s t-test.


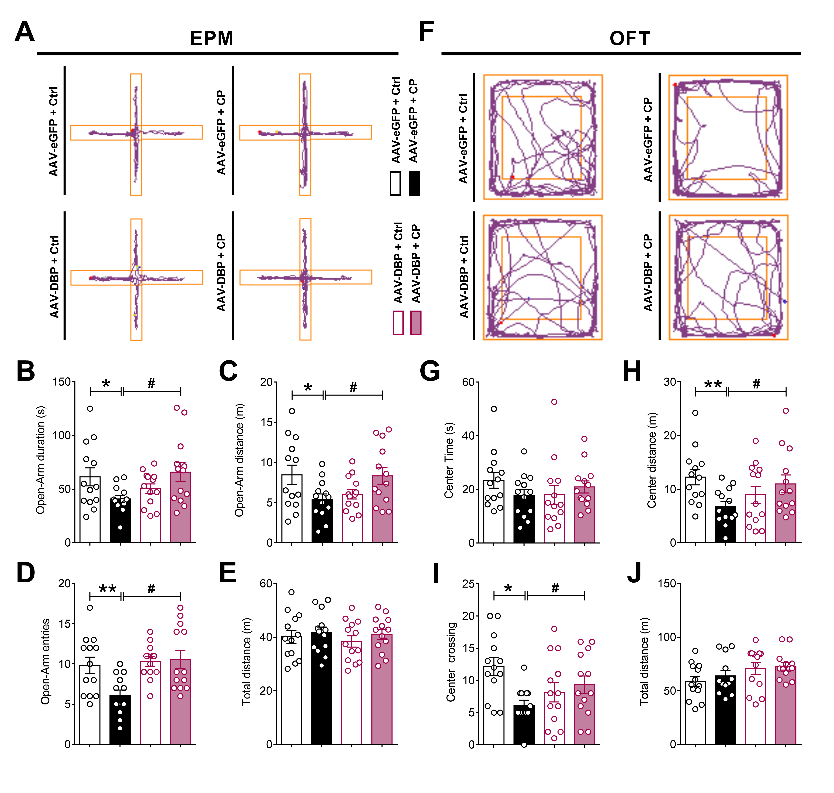


**Figure S6.** Microglial DBP signaling is required for anxiety-like behaviors induced by chronic periodontitis. A) The representative moving traces of Ctrl and CP mice treated with virus injection in the EPM. B-E) The statistical results of the open-arm duration (B), open-arm distance (C), open-arm enteries (D) and total distance (E) of Ctrl and CP mice treated with virus injection in EPM. n = 13 in each group. F) The representative moving traces of Ctrl and CP mice treated with virus injection in OFT. G-J) The statistical results of the center time duration (G), center distance (H), center crossing times (I) and total distance (J) of Ctrl and CP mice treated with virus injection in EPM. n = 13 in each group. All data are presented as the mean ± SEM. *p < 0.05 and **p < 0.01 vs. AAV-eGFP + Ctrl group, ^#^p < 0.05 vs. AAV-eGFP + CP group using two-way ANOVA.


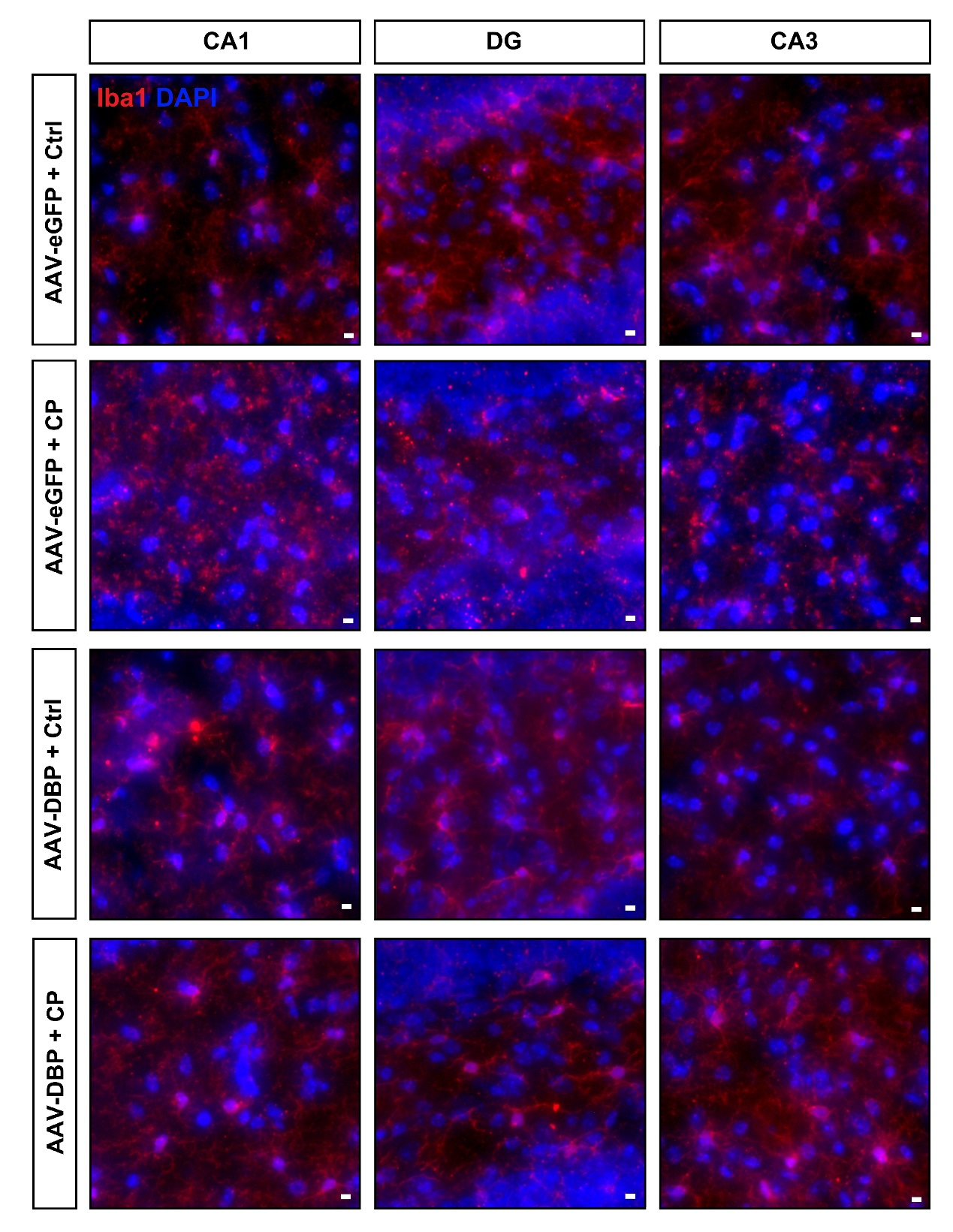


**Figure S7.** Effects of microglial DBP knockdown on the morphology of microglia in the hippocampus.

**
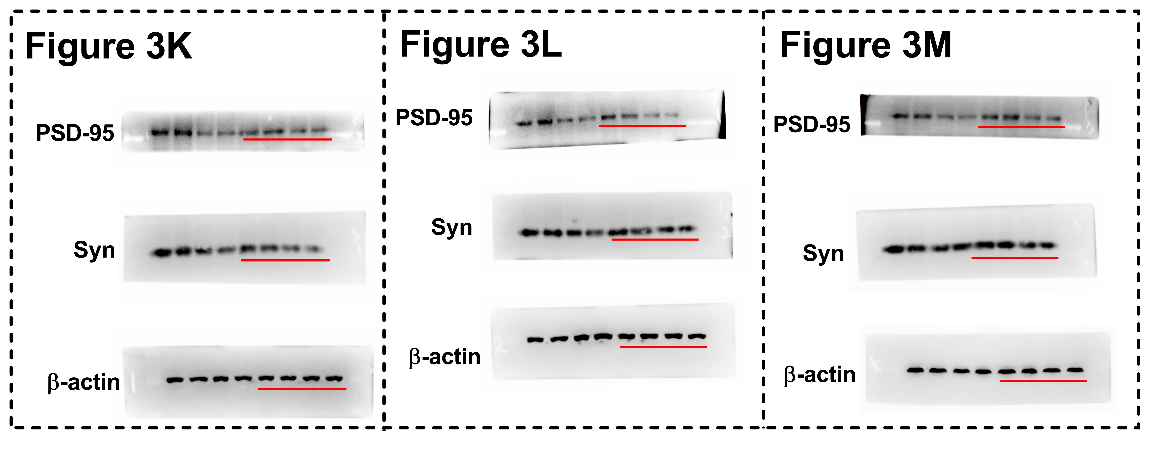
**

**Figure S8.** Full original images of Western blotting assays for Figure 3.

**
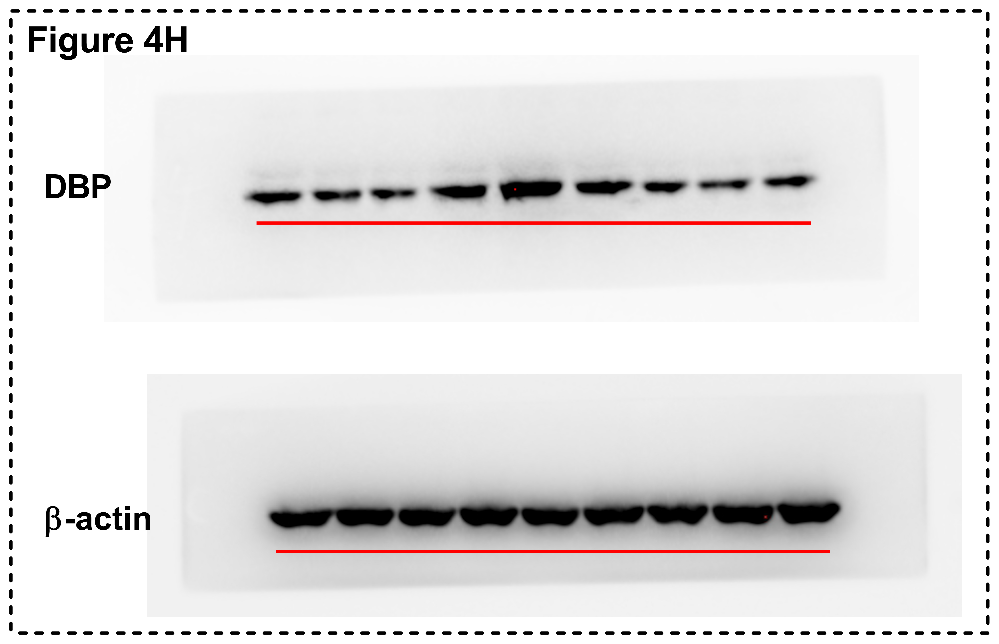
**

**Figure S9.** Full original images of Western blotting assays for Figure 4.

**
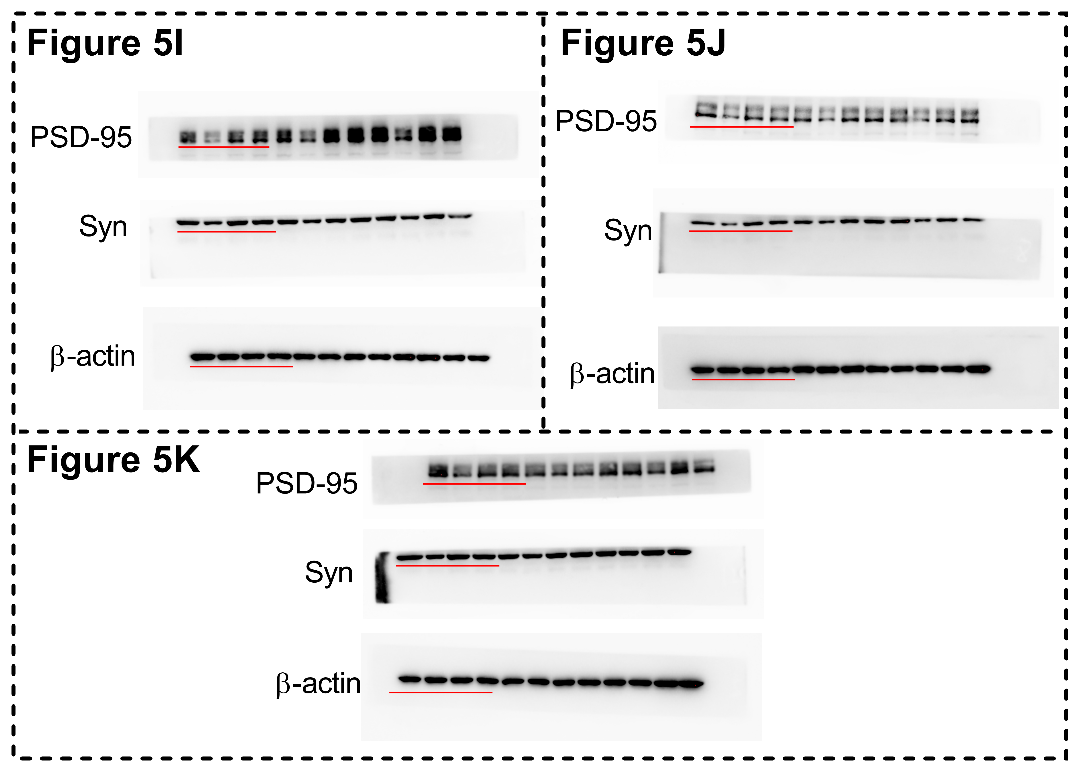
**

**Figure S10.** Full original images of Western blotting assays for Figure 5.

**
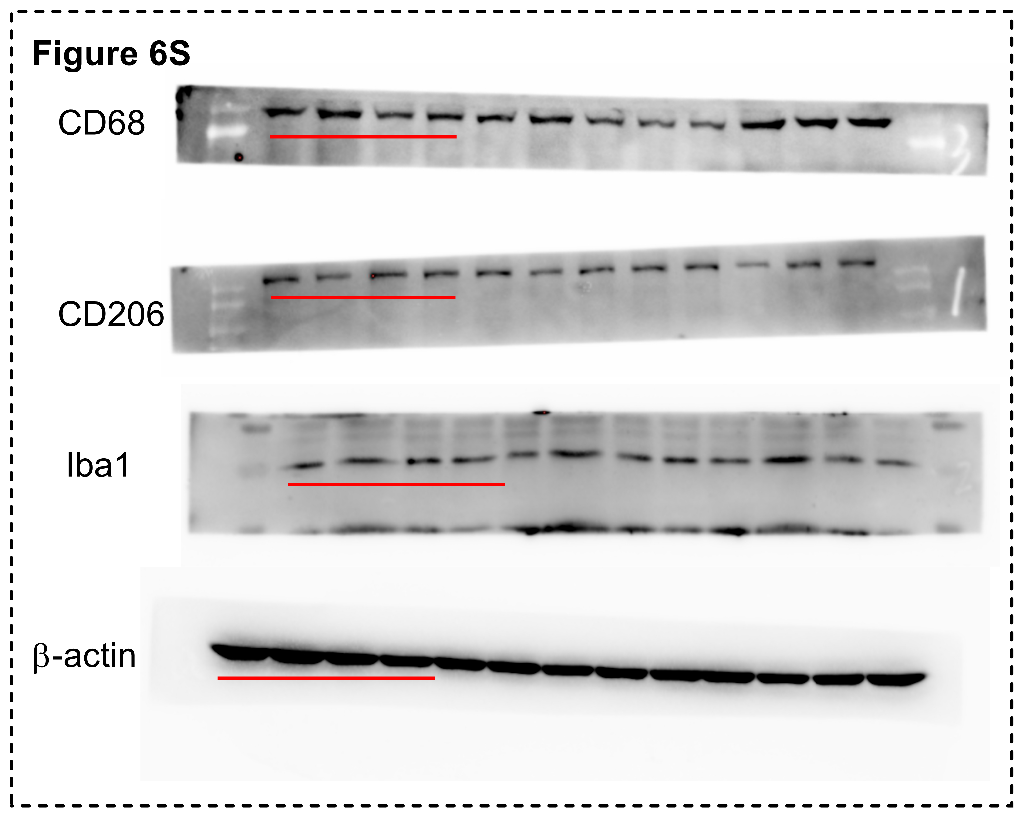
**

**Figure S11.** Full original images of Western blotting assays for Figure 6.

**
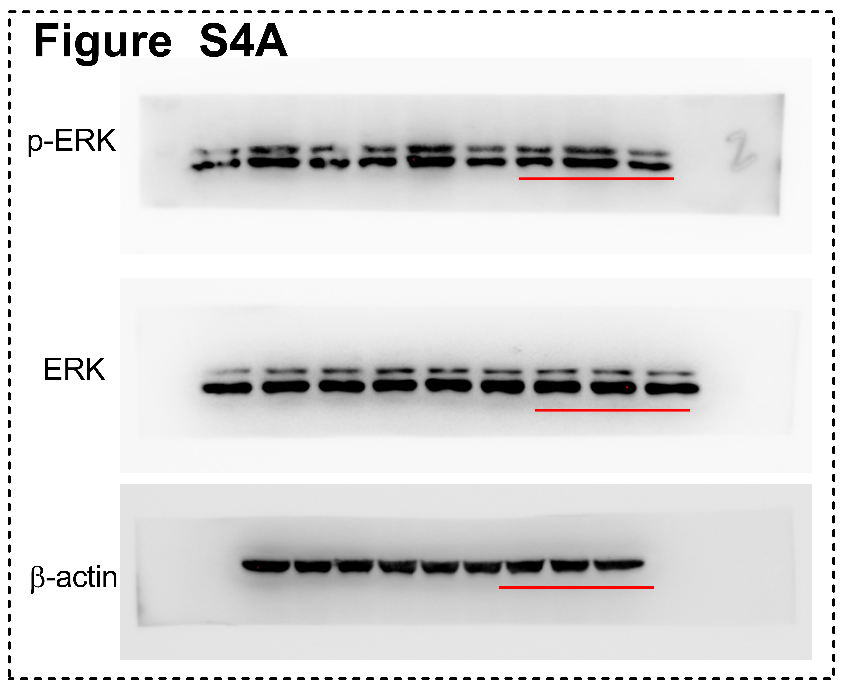
**

**Figure S12.** Full original images of Western blotting assays for Figure S4.


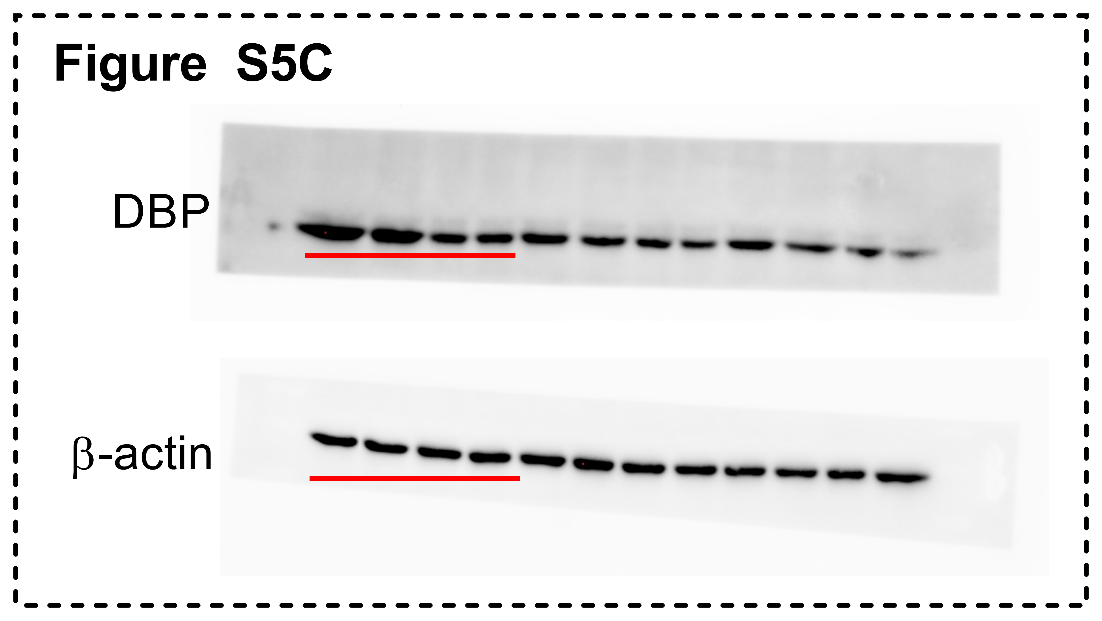


**Figure S13.** Full original images of Western blotting assays for Figure S5.

**Supplementary Tables**

**Table S1.** Information of antibodies used in Western blotting and immunofluorescence assays.

| **Antibodies** | **Manufacturer** | | **Identifier** |
| --- | --- | --- | --- |
| Rabbit polyclonal anti-PSD-95 | Millipore | Cat# MAB1596; RRID: AB_2092365 | |
| Rat monoclonal anti-BrdU | Abcam | Cat# ab6326; RRID: AB_305426 | |
| Rabbit monoclonal anti-NeuN | Abcam | Cat# ab177487; RRID: AB_2532109 | |
| Rabbit polyclonal anti-Doublecortin | Abcam | Cat# ab18723; RRID: AB_732011 | |
| Rabbit polyclonal anti-Ki67 | Abcam | Cat# ab15580; RRID: AB_7443209 | |
| Rabbit polyclonal anti-Synaptophysin | Abcam | Cat# ab16659; RRID: AB_443419 | |
| Rabbit monoclonal anti-DBP | Abcam | Cat# ab22824; RRID: AB_447320 | |
| Rabbit polyclonal anti-IBA1 | Abcam | Cat# ab153696; RRID: AB_2889406 | |
| Rabbit monoclonal anti-CD86 | Abcam | Cat# ab269587; RRID: AB_2943178 | |
| Rabbit monoclonal anti-CD206 | Cell Signaling Technology | Cat# 24595; RRID: AB_2892682 | |
| Mouse monoclonal anti-GFAP | Cell Signaling Technology | Cat# 3670; RRID: AB_561049 | |
| Mouse monoclonal anti-ERK | Cell Signaling Technology | Cat# 4695S; RRID: AB_390779 | |
| Mouse monoclonal anti-Phospho-ERK | Cell Signaling Technology | Cat# 4370S; RRID: AB_2315112 | |
| Mouse monoclonal anti-β-actin | Thermofisher Scientific | Cat# MA1-140; RRID: AB_2536844 | |
| Donkey anti-rabbit Alexa Fluor 594 | Thermofisher Scientific | Cat# A-21207; RRID: AB_141637 | |
| Donkey anti-rabbit Alexa Fluor 488 | Thermofisher Scientific | Cat# A-21206; RRID: AB_2535792 | |
| Donkey anti-mouse Alexa Fluor 594 | Thermofisher Scientific | Cat# A-11032; RRID: AB_2534091 | |
| Donkey anti-mouse Alexa Fluor 488 | Thermofisher Scientific | Cat# A-11029; RRID: AB_2534088 | |
| Goat anti-Rabbit IgG (H+L)  Secondary Antibody, HRP | Thermofisher Scientific | Cat# 31460; RRID: AB_228341 | |
| Mouse anti-Goat IgG (H+L)  Secondary Antibody, HRP | Thermofisher Scientific | Cat# 31400; RRID: AB_228370 | |
| Goat anti-Mouse IgG (H+L)  Secondary Antibody, HRP | Thermo Fisher Scientific | Cat# 31430; RRID: AB_228307 | |

**Table S2.** Oligonucleotide primers used for mRNA real time PCR.

| **Gene** | **Sense primer (5’ to 3’)** | | **Antisense primer (5’ to 3’)** |
| --- | --- | --- | --- |
| IL-1β- | GAAGGGCTGCTTCCAAACCT | TGATGTGCTGCTGCGAGATT | |
| TNF-α | CGGGCAGGTCTACTTTGGAG | ACCCTGAGCCATAATCCCCT | |
| IL-6 | CCCCAATTTCCAATGCTCTCC | CGCACTAGGTTTGCCGAGTA | |
| CXCL10 | ССGCTGCАACTGCAТCCATA | CAATGATCTCAACACGTGGGC | |
| iNOS | GCATCCCAAGTACGAGTGGT | GGTGCCCATGTACCAACCAT | |
| IL-4 | CCCCAGCTAGTTGTCATCCTG | CAAGTGATTTTTGTCGCATCCG | |
| IL-10 | CCTGGGTGAGAAGCTGAAGAC | CTTGTAGACACCTTGGTCTTGG | |
| IL-13 | CCTGGCTCTTGCTTGCCTT | GGTCTTGTGTGATGTTGCTCA | |
| TNF-β | GTCTGTGTATCCGGGACTTCA | TCTCCCTTACTGAGCAGGAAC | |
| Arg-1 | CGGGAGGGTAACCATAAGCC | CTTGGGAGGAGAAGGCGTTT | |
| CCL2 | TTAAAAACCTGGATCGGAACCAA | GCATTAGCTTCAGATTTACGGGT | |
| IFN-γ | TCAAGTGGCATAGATGTGGAAGAA | TGGCTCTGCAGGATTTTCATG | |
